# E2F function in muscle growth is necessary and sufficient for viability in *Drosophila*

**DOI:** 10.1038/ncomms10509

**Published:** 2016-01-29

**Authors:** Maria Paula Zappia, Maxim V. Frolov

**Affiliations:** 1 Department of Biochemistry and Molecular Genetics, University of Illinois at Chicago, 900 S Ashland Avenue, Chicago, Illinois 60607, USA

## Abstract

The E2F transcription factor is a key cell cycle regulator. However, the inactivation of the entire E2F family in *Drosophila* is permissive throughout most of animal development until pupation when lethality occurs. Here we show that E2F function in the adult skeletal muscle is essential for animal viability since providing E2F function in muscles rescues the lethality of the whole-body E2F-deficient animals. Muscle-specific loss of E2F results in a significant reduction in muscle mass and thinner myofibrils. We demonstrate that E2F is dispensable for proliferation of muscle progenitor cells, but is required during late myogenesis to directly control the expression of a set of muscle-specific genes. Interestingly, E2f1 provides a major contribution to the regulation of myogenic function, while E2f2 appears to be less important. These findings identify a key function of E2F in skeletal muscle required for animal viability, and illustrate how the cell cycle regulator is repurposed in post-mitotic cells.

## Abstract (web summary)

The transcriptional regulators E2F/Dp play a critical role in cell-cycle regulation, but it is unclear why E2F-deficient flies die. Here, the authors show this is linked to the function of E2F in adult *Drosophila* skeletal muscle, with the contribution of E2f1 being most important in post-fusion muscle.

## Graphical abstract

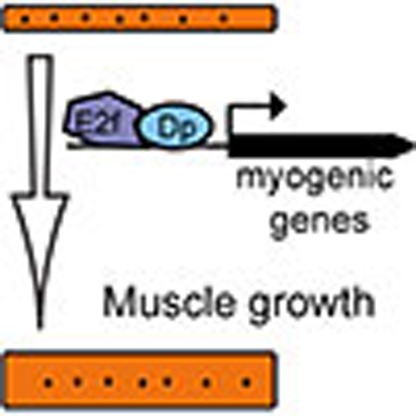

The E2F/DP heterodimeric transcription factors (hereafter referred as E2F) are the key cell cycle regulators and downstream targets of the Retinoblastoma tumour suppressor protein (pRB). Besides their prominent role in tumorigenesis, pRB and E2F are also critical in normal animal development; however, the phenotypes of the E2F and Rb knockout animals are not fully understood^1^,^2^,^3^. The arrangement of the *Drosophila* Rb pathway is simpler, yet highly analogous to that in mammals^4^. In flies, there are two E2Fs, an activator E2f1 and a repressor E2f2. Both *Drosophila* E2Fs heterodimerize with a single obligatory partner Dp, and, therefore, the loss of *Dp* eliminates all E2F activity and fully phenocopies the *E2f1 E2f2* double-mutant phenotype^5^,^6^. While most E2F targets are occupied by both E2f1 and E2f2, the relative contribution of each E2F family member to the overall level of expression of the target gene varies. The cell cycle E2F targets are regulated primarily by E2f1-dependent activation, while E2f2 represses the expression of differentiation genes and developmental genes in cycling cells^6^,^7^,^8^.

The streamlined version of the *Drosophila* E2F/Dp provides a unique opportunity to examine the impact of the complete genetic loss of E2F during development. Unexpectedly, *E2f1 E2f2* double-mutants progress normally throughout most of development, without any obvious defects in cell proliferation, differentiation or apoptosis^9^,^10^. Eventually, E2F-deficient animals die during pupal stages, and no *E2f1 E2f2* double-mutant adults could be recovered, suggesting that E2F is absolutely essential for animal viability. However, the studies on E2F-deficient animals thus far have failed to uncover a defect that could account for their lethality, and, therefore, the essential function of E2F in animal development remains unknown.

Here we show that E2F has an essential function in adult skeletal muscle, which is necessary and sufficient for animal viability. Although E2F is fully dispensable for proliferation of muscle progenitor cells, it is required for muscle growth and myofibrillogenesis. We show that E2F directly regulates the expression of late myogenic genes, with E2f1 providing the major contribution compared with E2f2. Overall, these results illustrate how a cell cycle transcriptional regulator is repurposed in post-mitotic cells to carry out an essential function during myogenic differentiation.

## Results

### Muscle-specific loss of Dp is lethal

**Figure 1:**
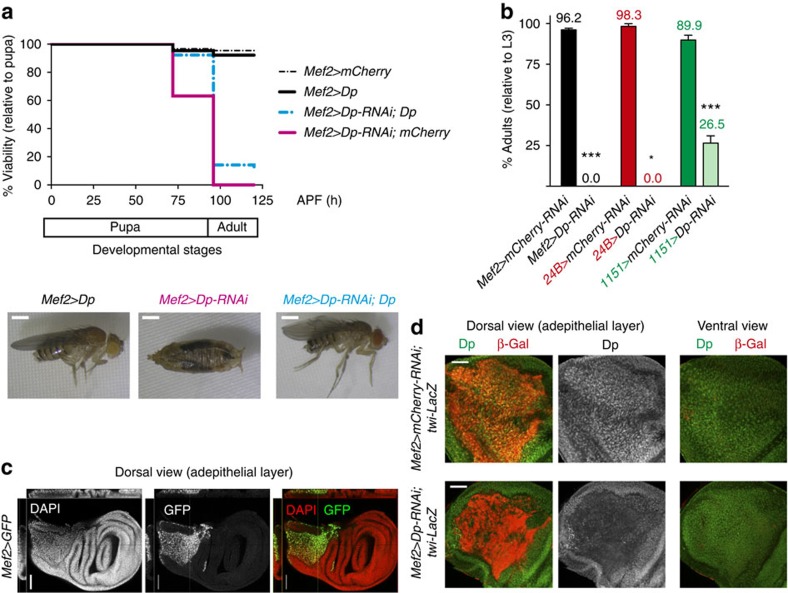
Muscle-specific inactivation of Dp results in lethality. (**a**) The number of viable flies was quantified throughout pupa development. The pan-muscular *Mef2-GAL4* driver was used to knockdown Dp by RNAi (*UAS-Dp-RNAi;Mef2-GAL4*, magenta line, *n*=654 flies). Re-expression of Dp partially rescues the lethality (*UAS-Dp-RNAi;Mef2-GAL4/UAS-Dp*, cyan-coloured dotted line, *n*=536 flies). The matching control flies are *Mef2-GAL4/UAS-mCherry-RNAi* (dotted black line, *n*=417 flies) and *Mef2-GAL4/UAS-Dp* (solid black line, *n*=523 flies). Data from three independent experiments were plotted. Representative images of adults and pharate pupa are shown at the bottom. Scale bar, 0.5 mm. (**b**) Lethality was quantified as the percentage of eclosed adults relative to the number of third instar larvae (L3). The GAL4 drivers *Mef2-GAL4* (black bar, *n*=239 and 173 flies), *24B-GAL4* (red bar, *n*=64 and 71 flies) and *1151-GAL4* (green bar, *n*=159 and 158 flies) were crossed either to *UAS-mCherry-RNAi* or *UAS-Dp-RNAi*. Mean±s.e.m. Data from at least two independent experiments were plotted, Mann–Whitney test. *P<0.05; ***P<0.001. (**c**) The expression of *Mef2-GAL4* is restricted to the adepithelial cells of late third instar larval wing discs. Several confocal images were taken in a z-stack. Orthogonal confocal cross-sections are presented on the top and left side of each image. Scale bar, 100 μm. (**d**) Wing discs of late third instar larvae stained with anti-Dp antibody (green) and labelled with *twi-lacZ* marker (β-Gal, red). Although Dp is localized in nuclei throughout the wing disc, Dp protein was specifically depleted only in the adult myoblast of *UAS-Dp-RNAi;Mef2-GAL4* (bottom panel) compared with control *Mef2-GAL4/UAS-mCherry-RNAi* (top panel). Two different confocal planes of the discs are shown: the dorsal view, which contains the adepithelial layer (left panel), and the ventral view (right panel). The experiment was repeated twice. Scale bar, 50 μm.

To identify the tissue in which E2F function is required for animal viability, we induced tissue-specific knockdown of Dp using RNA interference (RNAi) and the UAS/GAL4 system. The *UAS-Dp-RNAi* was crossed to a panel of GAL4 drivers, and animal viability was scored (Supplementary Table 1). Unexpectedly, the depletion of Dp using the myogenic *Mef2-GAL4* driver resulted in fully penetrant pupal lethality that mimicked the lethal phenotype of the whole-body *Dp* mutant (Fig. 1a,b and Supplementary Table 1). This result was validated using another muscle-specific GAL4 driver, *24B-GAL4*, and two independent nonoverlapping *UAS-Dp-RNAi* lines. Notably, pupal lethality in *Mef2>Dp*-RNAi animals was partially rescued by co-expression of a *UAS*-*Dp* transgene (Fig. 1a).

In *Drosophila*, the adult skeletal muscles are formed during pupal development^11^. The myoblasts, also known as adult muscle precursors, proliferate in the adepithelial layer at the dorsal proximal side of the wing imaginal disc that give rise to the adult flight muscles^12^. In late third instar larvae, these proliferating myoblasts can be visualized by the expression of Myocyte Enhancer Factor 2 (Mef2) and Twist bHLH (Twi) transcription factors^13^,^14^, using *Mef2-GAL4*-driven *GFP* (green fluorescent protein) and *twi-lacZ* markers, respectively (Fig. 1c,d). The efficiency of muscle-specific Dp knockdown was confirmed by staining wing imaginal discs of *Mef2>Dp-RNAi*; *twi-lacZ* animals with Dp and β-Gal antibodies. Dp was depleted specifically in myoblasts (β-Gal-positive) but not in the adjacent epithelial cells of the wing imaginal disc (β-Gal-negative; Fig. 1d).

Both *Mef2-GAL4* and *24B-GAL4* are broadly expressed in all muscle types throughout development. In contrast, *1151-GAL4* is expressed exclusively in adult muscle precursors, developing adult skeletal muscles and tendon cells for the jump muscles, while it is not expressed in larval, cardiac or visceral muscles^15^,^16^,^17^,^18^. Inactivation of Dp using *1151-GAL4* resulted in a threefold reduction in the number of eclosing adults with lethality occurring at the pupal stage (Fig. 1b and Supplementary Table 1). Notably, the *1151>Dp-RNAi* escapers were significantly defective in their ability to fly and jump (Supplementary Table 1), which indicates impaired adult skeletal muscle function and development^19^. Consistently, depletion of Dp with either *Mhc.F3-580-GAL4* or *Act88F-GAL4*, which are expressed in differentiating indirect flight muscles, resulted in similar flight/jump defects (Supplementary Table 1). Taken together, these results suggest an essential role for E2F in adult skeletal muscles, which is crucial for animal viability.

### Muscle-specific expression of Dp rescues *Dp*-mutant lethality

**Figure 2:**
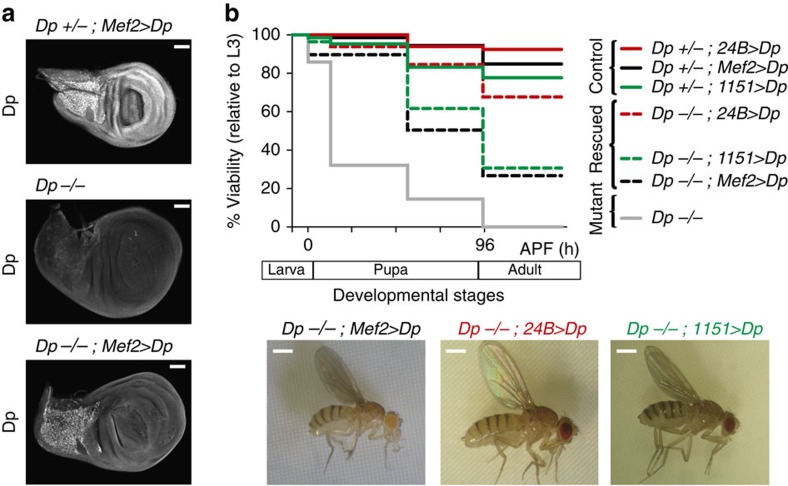
Expression of Dp in adult skeletal muscles rescues the lethality of the whole-body *Dp*^*−/−*^ null mutant animals. (**a**) Confocal sections of the adepithelial cells show that Dp expression is restricted to the adult myoblasts of the rescued animals (*Dp*^*a3*^*/Df(2R)Exel7124;Mef2-GAL4/UAS-Dp*, bottom panel), compared with the *Dp*-mutant (*Dp*^*a3*^/*Df(2R)Exel7124*, middle panel), and control (*Dp*^a3^*/+;Mef2-GAL4/UAS-Dp*, top panel). Representative images are shown. Scale bar, 0.5 μm. (**b**) The number of viable flies was quantified throughout development. *Dp* mutants, *Dp*^*a3*^/*Df(2R)Exel7124* (grey line, *n*=445), are lethal at mid-pupa stage. The lethality is partially rescued by overexpressing *UAS-Dp* in the muscle using either *Mef2-GAL4* (black dotted line, *n*=204 flies), *24B-GAL4* (red dotted line, *n*=65 flies) or *1151-GAL4* (green dotted line, *n*=72 flies). Control genotypes are *Dp*^a3^*/+; Mef2-GAL4/UAS-Dp* (black solid line, *n*=337 flies), *Dp*^a3^*/+;24B-GAL4/UAS-Dp* (red solid line, *n*=66 flies) and *1151-GAL4;Dp*^a3^*/+;UAS-Dp* (green solid line, *n*=113 flies). At least two independent experiments were performed using at least six replicates per genotype each. The genotypes *Dp*^*a3*^*/Df(2R)Exel7124;Mef2-GAL4/UAS-Dp*, *Dp*^*a3*^*/Df(2R)Exel7124;1151-GAL4/UAS-Dp*, and their respective controls were confirmed by single-fly PCR and by sequencing the *Dp*-mutant allele in individual flies. Representative images are shown at the bottom. Scale bar, 0.5 mm. See also Supplementary Fig. 1.

Results described above raise the possibility that the lethality in *Dp* mutants is due to the loss of Dp function in the muscle. If this is the case, then providing Dp function in muscles should be sufficient to rescue the whole-body *Dp* mutant, and permit the completion of development to adulthood. We expressed a *UAS*-*Dp* transgene in the *Dp* null mutant animals (*Dp*^*−/−*^) using the *Mef2-GAL4* driver. *Dp*^*−/−*^ animals were generated by crossing a known *Dp* null allele, *Dp*^*a3*^ (refs 5, 10), to a deficiency that removes the entire *Dp* gene, *Df(2R)Exel7124.* The muscle-specific expression of Dp in *Dp*^*−/−*^; *Mef2>Dp* animals was confirmed by staining wing discs with the Dp antibody. Dp expression was indeed restricted to adult myoblasts in the adepithelial layer and was completely absent in the imaginal disc (Fig. 2a).

Strikingly, while all *Dp* null mutants died during pupal development, 27% of *Dp*^*−/−*^; *Mef2>Dp* animals survived to adulthood (Fig. 2b). The rescued adult flies did not show any gross morphological defects, although they displayed a subtle rough eye phenotype and sterility, the latter likely reflects the known requirement of Dp in oogenesis^20^,^21^. Another muscle-specific driver, *24B-GAL4*, provided an even stronger rescue (68% of rescued *Dp*^*−/−*^; *24B>Dp* adults, Fig. 2b). The lethality of the *Dp* mutant was also rescued when Dp expression was limited to adult myoblasts and adult skeletal muscles using *1151-GAL4* (31% of rescued *Dp*^*−/−*^*;1151>Dp* adults, Fig. 2b). We confirmed that the rescue was not due to leaky expression of *Mef2-GAL4* and *24B-GAL4* drivers in non-related muscle tissues (Supplementary Fig. 1).

These results provide strong genetic evidence that the lethality in the *Dp* mutant was not caused by the requirement for E2F/Dp in the entire organism, but rather was due to the requirement of E2F/Dp function in adult skeletal muscle. Therefore, to understand why E2F/Dp is so important in this tissue, we examined its role during adult skeletal muscle development.

### Knockdown of Dp reduces the size of adult skeletal muscles

**Figure 3:**
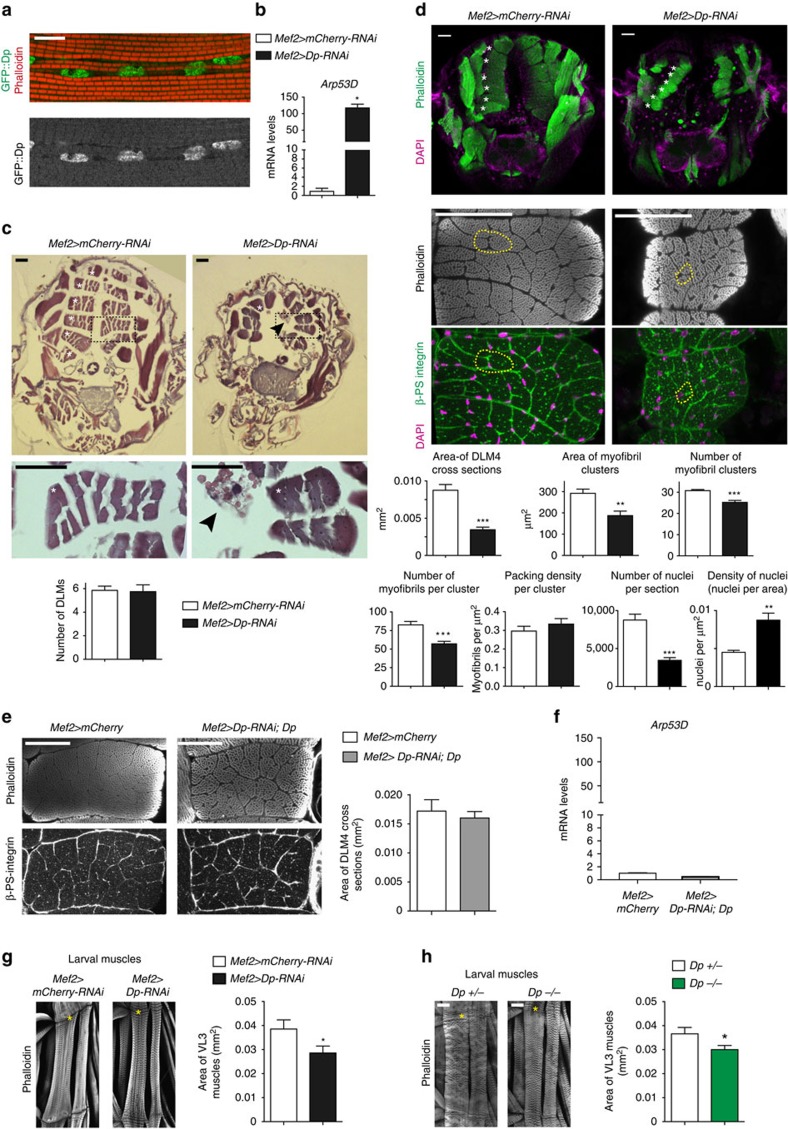
Loss of Dp reduces the size of larval and adult skeletal muscles. (**a**) Dp::GFP localizes in DLM nuclei of *Dp*^*GFP*^ pharates. Scale bar, 10 μm. See Supplementary Fig. 2. (**b**) *Arp53D* expression in flight muscle is de-repressed in Dp knockdown. RT–qPCR, mean±s.e.m., *N*=3 samples per genotype, Mann–Whitney test, *P<0.05. (**c**,**d**) Muscle size is reduced in Dp-depleted muscles. Transverse sections of pharate thoraces (ventral to the bottom). Each hemithorax contains six DLM (white asterisks, left panel). Scale bar, 50 μm. (**c**) Paraffin-embedded sections stained with haematoxylin and eosin. DLM4 (dashed black boxes) magnified at the bottom panel. Black arrowheads point the aggregates/clumps. Quantification of total number of DLMs per hemithorax, mean±s.d., *N*=21, 16 per genotype, Mann–Whitney test, *P*>0.05. (**d**) Sections stained with Phalloidin and 4,6-diamidino-2-phenylindole (DAPI; top panel). DLM 4 stained with Phalloidin (middle panel), β-PS integrin to mark the plasma membrane and DAPI (bottom panel). Cluster of myofibrils is outlined (yellow dashed line). Quantification of DLM4 cross-section area per fly (mm^2^). Area of myofibril clusters, total number of myofibril cluster, total number of myofibrils per cluster, myofibril density per cluster area (μm^2^), total number of nuclei and density of nuclei per DLM4 area (μm^2^) are quantified per DLM4, mean±s.e.m., *N*=8 per genotype, *t*-test with Welch's correction, **P<0.01; ***P<0.001. Three independent experiments. (**e**) Muscle size is rescued in thoraces of 2- to 5-day-old *UAS-Dp-RNAi;Mef2-GAL4/UAS-Dp* adults compared with *Mef2-GAL4/UAS-mCherry*. Sections stained with Phalloidin and β-PS integrin. Scale bar, 50 μm. Quantification of DLM4 cross-section area per fly (mm^2^), mean±s.e.m., *n*=8, 10 per genotype, *t*-test with Welch's correction, *P*>0.05. Two independent experiments. (**f**) *Arp53D* expression in flight muscles is rescued. Same genotype as in **e**. Mean±s.e.m., *N*=3 samples per genotype, Mann–Whitney test, *P*>0.05. (**g**,**h**) Third instar larval body wall muscle area is reduced in (**g**) Dp-depleted muscles and (**h**) in *Dp*^−/−^ mutant. Body wall muscles VL3 (asterisks) and VL4 stained with Phalloidin. Anterior is to the top. Scale bar, 50 μm. Quantification of VL3 muscle area, mean±s.e.m., unpaired *t*-test with Welch's correction, *P*<0.05. Three independent experiments. (**g**) *n*=10, 11, (**h**) *n*=12, 10 per genotype. Genotypes (**b**–**d**,**g**) *Mef2-GAL4/UAS-mCherry-RNAi* and *UAS-Dp-RNAi;Mef2-GAL4*, (**h**) *Dp*^*a3*^*/+* and *Dp*^*a3*^*/Df(2R)Exel7124*.

Staining with anti-Dp antibody and the *Dp[GFP]* reporter line^22^ revealed that Dp is broadly expressed in various muscle types (Supplementary Fig. 2A,B). High Dp levels were detected in the nuclei of larval body wall muscles, in cardiac and pericardial nuclei of the larval cardiac tube, in adult muscle precursors and in the indirect flight muscles of adult flies (Supplementary Fig. 2C–F) and pharate pupa (Fig. 3a). Since *Dp* mutants die at an early pupal stage, we examined *Mef2>Dp-RNAi* animals at the time point immediately preceding the lethal stage. The efficiency of Dp depletion was confirmed by measuring the expression of a known E2F target gene, *Arp53D*, which is de-repressed in response to E2F inactivation^7^ (Fig. 3b). The indirect flight muscles constitute more than 70% of the entire skeletal muscle mass and comprises two groups of muscles: dorsal longitudinal muscles (DLMs) and dorsal ventral muscles^23^. The architecture of DLMs was analysed by staining the transverse paraffin-embedded tissue sections with haematoxylin and eosin dyes. Most *Mef2>Dp-RNAi* pharates displayed six DLMs per hemithoraces (Fig. 3c), indicating that the main steps of muscle development are unaffected. However, each DLM cross-section area was significantly thinner compared with the control (white asterisks, Fig. 3c,d) suggesting that the overall muscle mass is reduced.

The structure of DLMs closely parallels that of vertebrate skeletal muscles. In DLMs, myofibrils are organized in clusters, with each cluster being surrounded by a membrane (dashed line in Fig. 3d). The area of each myofibril cluster is determined by the number of myofibrils and their packing density within the cluster. Thus, the small DLM size could be due to a reduction in the number of clusters or the cluster size. Frozen transverse tissue sections of thoraces were co-stained with Phalloidin and β-PS integrin antibody to visualize the myofibrils and the surrounding membrane, respectively (Fig. 3d). The overall structure and organization of DLM3 and DLM4 was examined. The number of clusters per DLM, the area of each cluster and the number of myofibrils per cluster were reduced in *Mef2>Dp-RNAi* compared with control. However, the packing density remained unaffected (Fig. 3d). Consistently, the number of nuclei per DLM was reduced. Importantly, the reduced muscle size was largely rescued by the re-expression of the *UAS-Dp* transgene in the Dp-depleted muscle of adult animals (Fig. 3e). As expected, *Arp53D* became fully repressed, confirming that Dp function is restored in *Mef2>Dp-RNAi;UAS-Dp* (Fig. 3f). Thus, we concluded that Dp-deficient muscles are smaller because of a reduction in the number of myofibrils per DLM without changes in muscle architecture.

To determine whether Dp depletion affects the size of other types of muscles in *Drosophila*, body wall preparations of third instar larvae were stained with phalloidin, and the size of the ventral longitudinal 3 (VL3) muscle was measured. As in the flight muscles, the Dp-depleted larval muscles were thinner (Fig. 3g). Importantly, the size of VL3 was reduced in *Dp*^*−/−*^ mutant to a similar extent to *Mef2>Dp-RNAi* larvae (Fig. 3h). Thus, we concluded that the reduced muscle size was specifically the result of Dp depletion in *Mef2>Dp-RNAi* larvae because it was also observed in mutant animals. However, the reduction in larval muscle size was relatively subtle, thus suggesting that E2F plays a minor role in larval skeletal muscle development in contrast to its requirement in adult skeletal muscles.

### Dp depletion does not alter myoblast proliferation or fusion

*Drosophila* muscle development occurs in a highly ordered manner that is tightly coordinated with the myogenic transcription programme. Adult myoblasts proliferate during larval stages within the adepithelial layer of the wing imaginal disc. In early pupae, myoblasts migrate and fuse into multinucleated myotubes. Fusion is completed by 36 h APF (after pupa formation), and, during the remainder of pupal development, DLMs increase in size because of the addition of myofibrils, and the assembly of the sarcomeres^23^,^24^,^25^. This late stage of myogenesis is characterized by a high level of expression of genes encoding structural and contractile proteins, which supports the increase in muscle mass. Thus, the final muscle size is determined by (1) the number of myoblasts and their fusion competence, (2) the proper assembly of myofibrils and (3) the ability of the muscle to grow. To understand why the loss of Dp results in reduced muscle size, we examined the effect of Dp depletion on each step of myogenesis.

**Figure 4:**
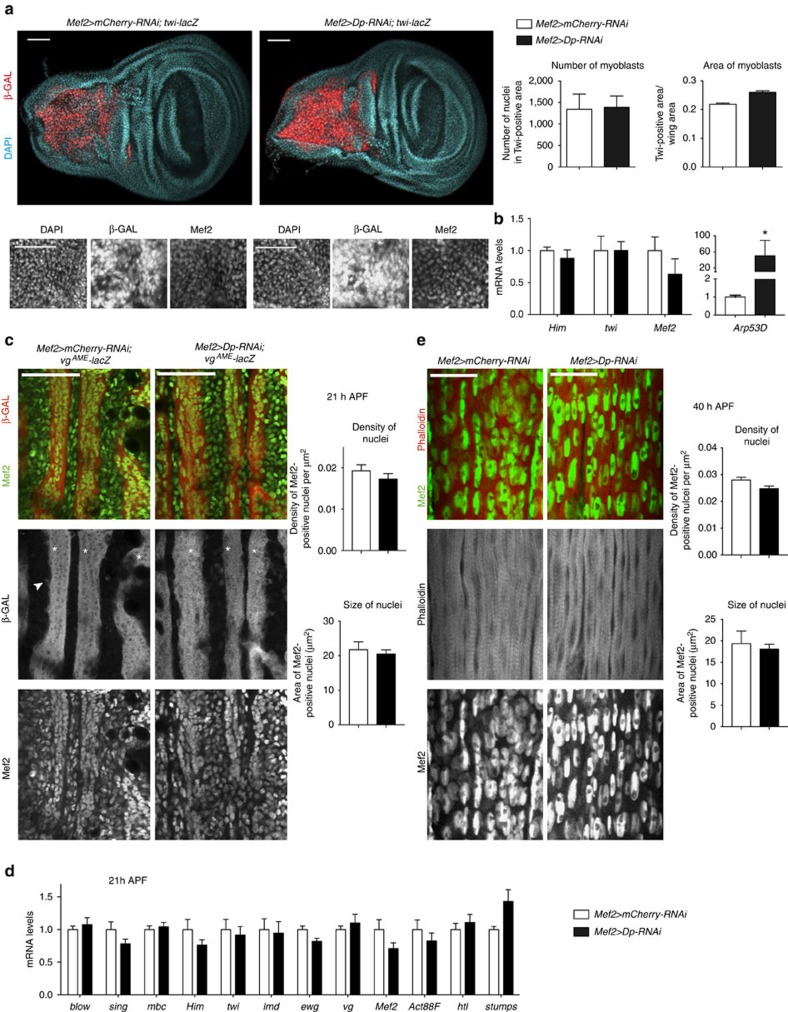
Loss of E2F does not affect myoblast proliferation or fusion at the onset of myotube formation. (**a**) Myoblast area and number are not altered. Confocal images of late third instar wing discs stained with anti-β-GAL (*twi-lacZ*), anti-Mef2 antibody and DAPI. Z-stack-projected images of adepithelial area were magnified. Quantification of total number of nuclei in Twi-positive area using DAPI staining, and Twi-positive area relative to total wing area, mean±s.e.m., *n*=14, 10 wing discs per genotype, *t*-test with Welch's correction, *P*>0.01. Two independent experiments. (**b**) The expression of myoblast-specific genes in wing discs is not affected. Expression of *twi*, *Holes-in-muscles* (*Him*) and *Mef2* genes quantified by RT–qPCR. *Arp53D* was de-repressed in Dp-depleted wing discs. Mean±s.e.m., *N*=3 and 5 samples per genotype, two-way analysis of variance (ANOVA), *P*>0.05 (left panel) and Mann–Whitney test, *P<0.05 (right panel). (**c**) The onset of myotube differentiation proceeds normally. Developing DLMs (asterisks) at 21 h APF stained with the reporter *vg*^*AME*^*-lacZ* to mark the onset of myotube formation and anti-Mef2 antibody. Arrowhead points to a fusion event. Anterior is to the top. Quantification of the total number of Mef2-positive nuclei per image (μm^2^), the area of Mef2-positive nuclei (μm^2^), mean±s.e.m., *N*=9 thoraces per genotype, *t*-test with Welch's correction, *P*>0.05. Three independent experiments. (**d**) The expression of myogenic genes in developing flight muscles at 21 h APF is normal. The genes measured are *blown fuse* (*blow*), *singles bar* (*sing*), *myoblast city* (*mbc*), *Holes-in-muscles* (*Him*), *twist* (*twi*), *lame duck* (*lmd*), *erect wing* (*ewg*), *vestigial* (*vg*), *Mef2*, *Act88F*, *htl* and *stumps*. mean±s.e.m., *N*=4 independent samples per genotype, two-way ANOVA, *P*>0.05. (**e**) Myoblast fusion to developing DLMs is not grossly altered. Developing DLMs at 40 h APF stained with Phalloidin to mark early myofibrils and anti-Mef2 antibody. Anterior is to the top. Quantification of the total number of Mef2-positive nuclei per area (μm^2^), the area of Mef2-positive nuclei (μm^2^), Mean±s.e.m., *N*=8 thoraces per genotype, *t*-test with Welch's correction, *P*>0.01. Two independent experiments. Genotypes are *Mef2-GAL4/UAS-mCherry-RNAi* and *UAS-Dp-RNAi;Mef2-GAL4*. Scale bar, 50 μm (**a**–**c**), and 20 μm (**e**).

We began by analysing proliferation in Dp-depleted adult myoblasts. The adult myoblasts within the adepithelial layer of *Mef2>Dp-RNAi* and control discs were blindly quantified using *twi-lacZ* reporter and Mef2 antibody as myoblast-specific markers (Fig. 4a). The number of Dp-depleted myoblasts and the corresponding area were indistinguishable from that of control (Fig. 4a). Consistently, the expression of genes encoding myogenic regulators, *twi*, *Holes-in-muscles* (*Him*) and *Mef2*, was not significantly different in Dp-depleted adult myoblasts compared with control (Fig. 4b). Dp depletion was efficient because the E2F target *Arp53D* was strongly de-repressed (Fig. 4b). Thus, the small size of Dp-deficient adult muscles was not because of a reduction in the pool of adult myoblasts.

Next, developing myotubes were visualized with the *vestigial adult muscle enhancer* (*vg*^*AME*^*-lacZ*) reporter^26^ at 21 h APF when wild-type migrating myoblasts are undergoing fusion. Dp-depleted myotubes properly activated *vg*^*AME*^*-lacZ* expression (white asterisks, Fig. 4c) and, in several cases, expression of *vg*^*AME*^*-lacZ* was detected in individual myoblasts immediately before fusion similarly to that observed in control (arrowhead, Fig. 4c). Counting the number of Mef2-positive nuclei in the nascent myotubes and surrounding myoblasts revealed a subtle, yet not statistically significant, reduction in *Mef2>Dp-RNAi* (Fig. 4c). These results suggest that at 21 h APF both migration and fusion proceeded normally.

This conclusion was corroborated using quantitative reverse transcriptase–PCR (qRT–PCR). The expression of genes encoding fusion proteins (*blow*, *sing*, *mbc*), myogenic regulators (*Him*, *twi*, *lame duck* (*lmd*), *Mef2, erect wing* (*ewg*), *vg*), muscle progenitor specification genes (*heartless* (*htl)*, *stumps*) and the structural gene *Actin 88F* (*Act88F)* was normal at 21 h APF in Dp-depleted muscles (Fig. 4d).

To determine whether all migrating Dp-depleted myoblasts properly completed fusion to developing DLMs, we examined DLMs at 40 h APF, when fusion is complete. The multinucleated DLMs were stained with Phalloidin to reveal the overall architecture, and with Mef2 antibody to mark individual nuclei. The number of nuclei in Dp-depleted DLMs was slightly but not significantly reduced (Fig. 4e). Thus, knockdown of Dp does not affect the overall pool of myoblasts or their ability to migrate and fuse properly.

### Growth is defective in Dp-depleted adult skeletal muscles

The development of the indirect flight muscles culminates in myofibrillogenesis, during which actin and myosin filaments are assembled to form myofibrils, which in turn contain the highly ordered repetitive contractile units known as sarcomeres. Sarcomeres are composed of parallel thick and thin filaments. Thin filaments from neighbouring sarcomeres are crosslinked in the Z-disc, and thick filaments are crosslinked in the M-line. The indirect flight muscles express specific proteins, such as Flightin (Fln), and muscle-specific isoforms of structural proteins, such as Act88F and Troponin C isoform 4 (refs 27, 28, 29). Myofibrillogenesis is accompanied by a dramatic increase in muscle size. The robust expression of muscle structural genes is needed to support muscle growth during the remainder of pupal development.

**Figure 5:**
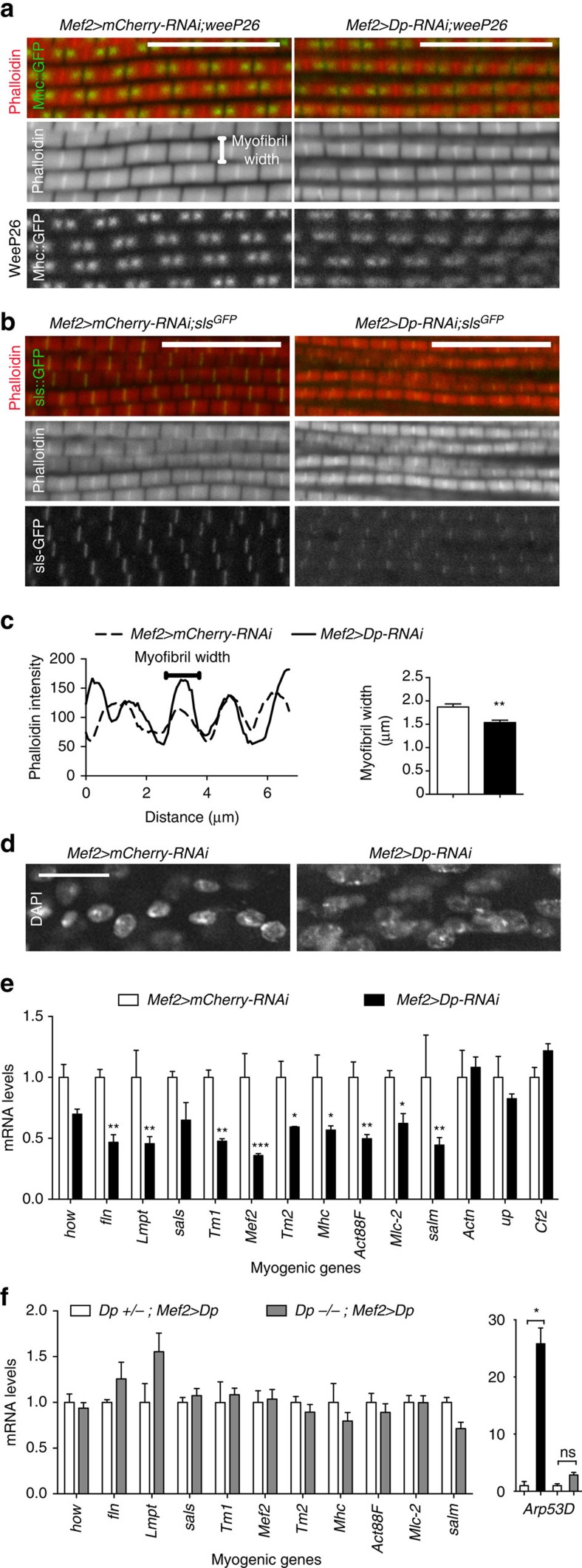
E2F controls myofibrillogenesis in flight muscles by regulating myogenic gene expression. (**a**–**c**) Myobrils and sarcomeric proteins are slightly misassembled in Dp-depleted muscles at the pharate stage. Confocal images of DLMs in a sagittal view stained with Phalloidin (red) in a GFP-tagged splice variant Mhc-IFM19 background (*weeP26*), to mark A-band structure (**a**), or in an Sls-GFP background to mark Z-band structure (**b**). Scale bar, 5 μm. (**c**) Profile plot of phalloidin intensity over distance. A line perpendicular to myofibril orientation was drawn to determine the number of myofibrils per distance. Quantification of myofibril width (μm), mean±s.e.m., *N*=8 and 7 hemithoraces per genotype, *t*-test with Welch's correction, **P<0.01. (**d**) Nuclei are enlarged in Dp-depleted muscles. Confocal images of DLMs in a sagittal view stained with DAPI. Scale bar, 20 μm. (**e**,**f**) Muscle-specific gene expression is compromised in Dp-depleted flight muscles at the pharate stage, and rescued by overexpressing Dp only in muscles of Dp-mutant background. Muscle genes are *how*, *fln*, *Lmpt*, *sals*, *Tm1*, *Mef2*, *Tm2*, *Mhc*, *Act88F, Mlc2*, *salm*, *Actn*, *Cf2* and *upheld* (*up, TpnT* or *wupB*). Mean±s.e.m., *N*=3 independent samples per genotype, two-way ANOVA (**e**), Kruskal–wallis test (**f**), *P<0.05, **P<0.01, ***P<0.001. Genotypes are (**a**–**e**) *Mef2-GAL4/UAS-mCherry-RNAi* (left panel, white bar) and *UAS-Dp-RNAi;Mef2-GAL4* (right panel, black bar), and (**f**) *Dp*^a3^*/+; Mef2-GAL4/UAS-Dp* (white bar) and *Dp*^*a3*^/*Df(2R)Exel7124;Mef2-GAL4/UAS-Dp* (grey bar).

The DLMs were dissected from pharate adults (96 h APF), and longitudinal sections were stained with phalloidin to visualize myofibril organization. Dp-depleted muscle showed a mild disorganization in the myofibril structure. The assembly of sarcomere was assessed using a GFP-tagged Mhc isoform, weeP26, that localizes to two foci flanking the M-line at the core of the sarcomere^30^. The localization of weeP26 in Dp-depleted muscles was more diffuse and the intensity of the signal was reduced compared with control (Fig. 5a). The Z-line constitutes a critical anchoring point for thin filaments and can be visualized using the Sls-GFP reporter^30^. Notably, the pattern of Z-lines in Dp-depleted DLMs was less definitive than in control (Fig. 5b). In addition, myofibril width was significantly reduced in Dp-depleted DLMs, as shown by the plot profile of phalloidin intensity over distance (Fig. 5c), suggesting an impaired recruitment of thick filaments. Interestingly, nuclei were larger in Dp-depleted muscles than in control (Fig. 5d). Thus, Dp-depleted muscles formed sarcomeres properly; however, the sarcomere structure was less pronounced, which might result from stoichiometric imbalance among sarcomere components^31^.

Since myofibrillogenesis and the accompanying muscle growth depend on robust expression of structural genes, we examined the level of myogenic transcripts in flight muscles of pharate using qRT–PCR. The expression of genes encoding structural and contractile proteins, such as *Myosin heavy chain* (*Mhc*), *fln*, *Tropomyosin 1* (*Tm1*), *Tropomyosin 2* (*Tm2*), *Myosin light chain 2* (*Mlc2*), *sarcomere length short* (*sals*) and *Act88F*, and myogenic regulators, such as *held out wings* (*how*), *Limpet* (*Lmpt*), *Myocyte enhancer factor 2* (*Mef2*) and *spalt major* (*salm*), were markedly reduced (Fig. 5e). We confirmed the decreased expression of these genes with an independent *UAS-Dp-RNAi* transgene (*Mef2>Dp-RNAi-TRiP*, Supplementary Fig. 3A). Importantly, the defect in gene expression was fully rescued by muscle-specific re-expression of the *UAS-Dp* transgene in the *Dp*^−/−^ mutant background (*Dp*^*−/−*^; *Mef2>Dp*, Fig. 5f, grey box) and was partially rescued in the *Dp-RNAi* background (*Mef2>Dp-RNAi*;*UAS-Dp*, Supplementary Fig. 4). In each experiment, we monitored the expression of *Arp53D* to ensure the efficient depletion of Dp in RNAi experiments and the rescue following Dp re-expression (Supplementary Fig. 3B and Figs 3f and 5f). Not every structural gene was reduced following Dp knockdown, such as *upheld* (or *Troponin T)* and *α-actinin* (*Actn*; Fig. 5e). Similarly, the myogenic transcription factor *Chorion factor 2* (*Cf2*), which represses the expression of *Act88F* in indirect flight muscles^32^, was unaffected (Fig. 5e).

These results suggest that the loss of Dp does not alter muscle development, but rather affects the growth of muscles, which is likely to be due to the reduced expression of a subset of late myogenic genes.

### Mitochondria are abnormal in Dp-depleted muscles

**Figure 6:**
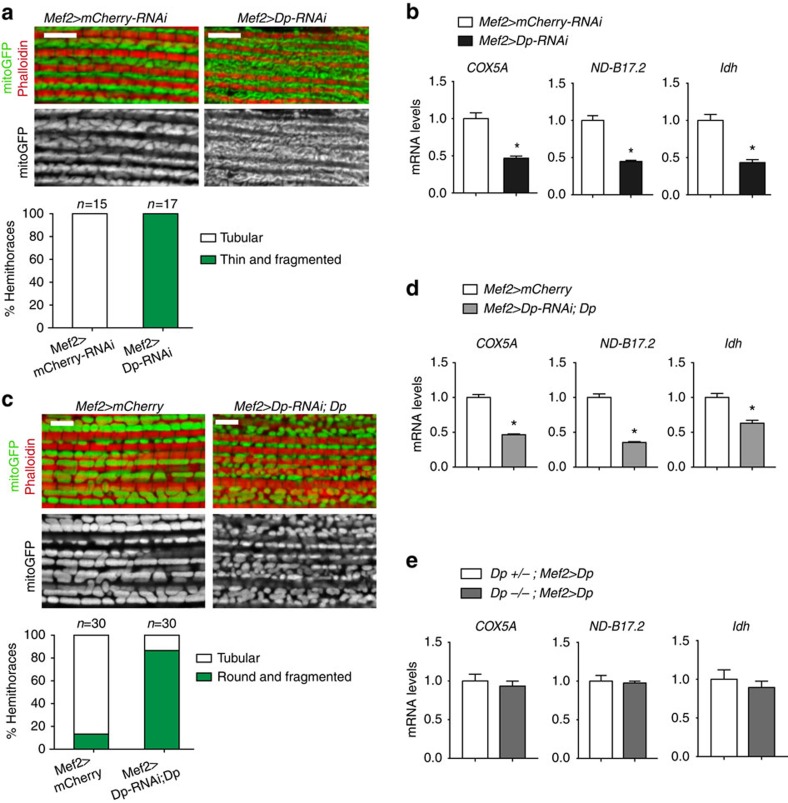
Mitochondria are abnormal in Dp-depleted muscles. The structure of mitochondria (**a**) and the expression of nuclear-encoded mitochondrial genes (**b**) are compromised in Dp-depleted muscles at the pharate stage. Although mitochondrial structure (**c**) and gene expression (**d**) are not fully reversed in the partially rescued adult flies (2- to 5-day-old), gene expression is fully restored in the rescued pharates that express Dp in *Dp*-mutant background (**e**). (**a**,**c**) Confocal images of DLMs stained with Phalloidin (red) and mitoGFP (green) to label mitochondria in a sagittal view. Scale bar, 5 μm. Quantification of hemithoraces displaying (**a**) round/tubular or thin/fragmented mitochondria shape, *N*=15 and 17 hemithoraces per genotype, (**c**) tubular or round/fragmented mitochondria shape, *n*=30 hemithoraces per genotype. Two independent experiments were performed. (**b**,**d**,**e**) The expression of the nuclear-encoded mitochondrial genes *Cytochrome c oxidase subunit 5A* (*Cox5A*, also known as *CoVa*), *NADH dehydrogenase* (*ubiquinone*) *B17.2 subunit* (*ND-B17.2*, *CG3214*) and *Isocitrate dehydrogenase* (*Idh*, *CG6439*), quantified using RT–qPCR in indirect flight muscles of pharate pupae (**b**,**e**) and adult flies (**d**). Mean±s.e.m., *N*=3 independent samples per genotype, Mann–Whitney test, *P<0.05. Genotypes are (**a**,**b**) *Mef2-GAL4/UAS-mCherry-RNAi* (left panel and white bar) and *UAS-Dp-RNAi;Mef2-GAL4* (right panel and black bar), (**c**,**d**) *Mef2-GAL4/UAS-mCherry* (left panel and white bar) and *UAS-Dp-RNAi;Mef2-GAL4/UAS-Dp* (right panel and grey bar), and (**e**) *Dp*^a3^*/+; Mef2-GAL4/UAS-Dp* (white bar) and *Dp*^*a3*^/*Df(2R)Exel7124;Mef2-GAL4/UAS-Dp* (grey bar).

During DLM growth, mitochondria undergo extensive fusion, increase in size^33^ and become large and tubular as revealed by *mitog*FP marker (Fig. 6a). Dp-depleted muscles failed to properly remodel mitochondria, which remained thin and elongated, resembling mitochondria at earlier time points of muscle development^33^. E2F/Dp was shown to regulate mitochondrial function by directly controlling the expression of mitochondria-associated genes in the eye^34^. In Dp-depleted muscle, the expression of mitochondria-associated E2F target genes, *COX5A*, *ND-B17.2* and *Idh* (ref. 34), were downregulated (Fig. 6b). Thus, the role of E2F in the regulation of mitochondria is conserved in *Drosophila* skeletal muscle.

We analysed mitochondrial morphology in rare *Mef2>Dp-RNAi;UAS-Dp* animals that were rescued to adulthood by muscle-specific expression of Dp (Fig. 1a) and no longer displayed the muscle growth defect (Fig. 3e). Interestingly, the mitochondrial network in *Mef2>Dp-RNAi;UAS-Dp* adults remained abnormal (Fig. 6c). Consistent with persistent mitochondrial defects, the expression of mitochondria-associated genes also remained low (Fig. 6d). These data suggest that both the muscle growth defect and the lethality of *Mef2>Dp-RNAi* animals can be rescued, at least partially, without fully suppressing the mitochondrial defect. Notably, in the *Dp*^*−/−*^ mutants that were rescued to adulthood by expression of *UAS-Dp* with *Mef2-GAL4*, the expression of mitochondria-associated genes was fully restored (Fig. 6e). The rescue of mitochondria network correlated with animal viability rescue, as ∼30% of *Dp*^*−/−*^*; Mef2>Dp* reached the adult stage versus 10% of *Mef2>Dp-RNAi; Dp* (Figs 1a and 2b). Thus, the rescue of muscle growth defects and mitochondrial function is needed in *Dp*-deficient animals to survive to adulthood.

### E2F directly regulates the expression of late myogenic genes

**Figure 7:**
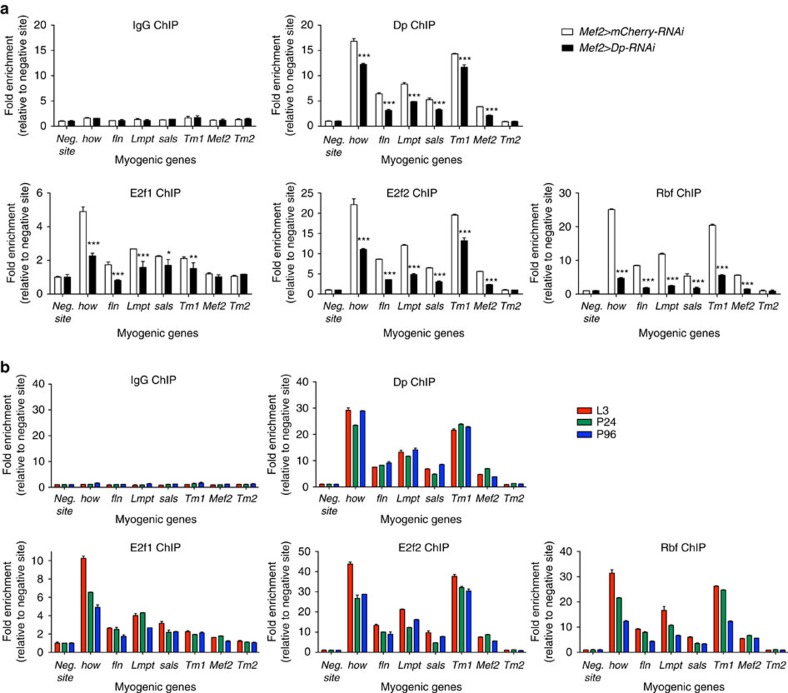
The E2F/Rb pathway directly controls myogenic gene expression throughout development. E2f1, E2f2, Dp and Rbf proteins are enriched upstream several myogenic genes in a Dp-dependent manner (**a**), and throughout development (**b**). Chromatin from pharate pupae (P96, **a**,**b**), third instar larvae (L3, **b**) and early pupae (P24, **b**) were immunoprecipitated with antibodies against Dp (top right panel), E2f1 (bottom left panel), E2f2 (bottom middle panel) or Rbf (bottom right panel), and compared with nonspecific control antibodies (top left panel). Muscle genes are *how*, *fln*, *Lmpt*, *sals*, *Tm1*, *Mef2* and *Tm2*. The negative site does not contain predicted E2F-binding sites. The qPCR data are shown as fold enrichment relative to the negative site for each ChIP sample. Mean±s.d., *n*=2 replicates, two-way ANOVA, *P<0.05, **P<0.01, ***P<0.001. Genotypes are (**a**) *Mef2-GAL4/UAS-mCherry-RNAi* (white bar) and *UAS-Dp-RNAi;Mef2-GAL4* (black bar), and (**b**) *Mef2-GAL4/UAS-mCherry-RNAi*.

To investigate the role of the E2F/Rb pathway in regulating the expression of late myogenic genes, we selected genes that were downregulated in Dp-deficient muscles and contained putative E2F-binding sites in the proximity of the transcription start site. Chromatin was isolated from pharate pupa and was subjected to chromatin immunoprecipitation (ChIP)–qPCR. Significantly, upstream regions of myogenic genes, such as *how*, *fln*, *Lmpt*, *sals*, *Tm1* and *Mef2*, were specifically enriched with E2f1, E2f2, Dp and Rbf antibodies in comparison with a nonspecific antibody (IgG, Fig. 7a). No enrichment was detected for the negative site, which did not contain predicted E2F-binding sites. Some myogenic genes, such as *Tm2*, did not show any enrichment upstream the transcription start site (Fig. 7a), suggesting that either their expression is indirectly regulated by E2F or E2F binds to a different region from the one tested using ChIP–qPCR.

Next, we performed ChIP–qPCR using chromatin isolated from *Mef2>Dp-RNAi* pharate adults, in which Dp was depleted exclusively in muscles, while the remainder of the animals expressed normal levels of endogenous Dp. The muscle-specific depletion of Dp resulted in significantly reduced enrichment of E2f1, E2f2, Dp and Rbf on the myogenic genes (Fig. 7a). This suggests that E2F/Dp is present as a complex, and that the recruitment of Rbf is E2F-dependent.

Since the expression of the contractile and structural genes is restricted to a narrow window during mid to late pupal development, we examined the occupancy of E2F/Dp/Rbf on these genes during different time points in development. Chromatin was isolated from third instar larva, early pupa (24 h APF) and pharate (96 h APF) animals. Notably, E2f1, E2f2, Dp and Rbf were highly enriched at these promoters even before the activation of *fln, Lmpt and Tm1* gene expression (Fig. 7b). These results suggest that, although E2F/Dp is required for the expression of several myogenic genes, it is insufficient to activate their expression by itself. Therefore, other myogenic transcription factors are operating at this point to induce their expression.

### E2f1 provides a major contribution to skeletal muscle growth

**Figure 8:**
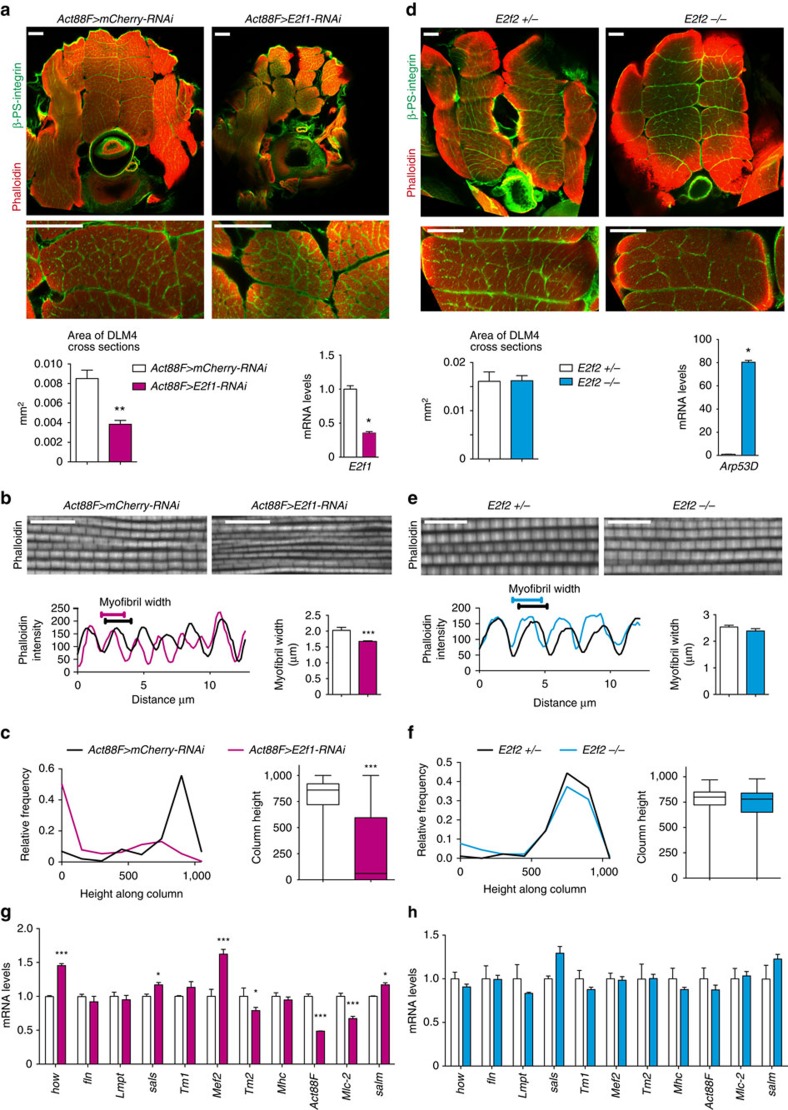
E2f1 is a major contributor to muscle growth and function in post-fused indirect flight muscles. (**a**,**d**) Muscle size is reduced in E2f1-depleted muscles (*Act88F>E2f1-RNAi*), whereas *E2f2* mutant muscles are normal. Transverse sections stained with Phalloidin and β-PS integrin (ventral to the bottom, top panel). DLM4 is magnified (middle panel). Scale bar, 50 μm. Representative images are shown. Quantification of DLM4 cross-section area per fly (mm^2^), mean±s.e.m., Mann–Whitney test, **P<0.01. (**a**) *n*=6, (**d**) *n*=3,4 flies per genotype. Expression of *E2f1* (**a**, bottom right panel) and *Arp53D* (**b**, bottom right panel) in flight muscles measured by RT–qPCR. Mean±s.e.m., *N*=3 independent samples per genotype, Mann–Whitney test, *P<0.05. (**b**,**e**) Myofibril width is slightly reduced in *Act88F>E2f1-RNAi*, whereas in *E2f2* mutant it is normal. Confocal images of DLMs in a sagittal view stained with Phalloidin. Scale bar, 10 μm. Profile plot of phalloidin intensity over distance (bottom left panel). A line perpendicular to myofibril orientation was drawn to determine the number of myofibrils per distance. Quantification of myofibril width (μm; bottom right panel), mean±s.e.m., Mann–Whitney test, **P<0.01. (**b**) *n*=7,8, (**e**) *n*=6 hemithoraces per genotype. (**c**,**f**) Flight performance is poor in *Act88F>E2f1-RNAi* adult flies, whereas in *E2f2* mutant it is normal. Female flies were flipped into a mineral-coated column, and the frequency of flies landing over the height of the column was scored. Box plots (Min to Max), Mann–Whitney test, ***P<0.001. Three independent experiments were conducted. (**c**) *n*=147,205, (**f**) *n*=90,91 flies per genotype. (**g**,**h**) Muscle-specific gene expression is compromised in *Act88F>E2f1-RNAi* flight muscles of pharate, whereas no gross alteration is found in *E2f2* mutant. Genes are *how*, *fln*, *Lmpt*, *sals*, *Tm1*, *Mef2*, *Tm2*, *Mhc*, *Act88F, Mlc2* and *salm*. Mean±s.e.m., *N*=3 independent samples per genotype, two-way ANOVA, *P<0.05, ***P<0.001. Genotypes (**a**–**c**,**g**) *Act88F-GAL4;UAS-mCherry-RNAi* and *Act88F-GAL4;UAS-E2f1-RNAi*, (**d**–**f**,**h**) *E2f2*^*c03344*/+^ and *E2f2*^*c03344*^/*E2f2*^*76Q1*^. Pharate (P96 h APF, **a**,**b**,**g**) and adult flies (**c**,**d**–**f**,**h**).

During cell proliferation, E2f1 and E2f2 act antagonistically to each other, with E2f1 being an activator and E2f2 acting as a repressor^7^,^9^. The loss of *E2f1* blocks cell proliferation and results in larval lethality^35^,^36^; however, this defect is rescued by the *E2f2* mutation as *E2f1 E2f2* double mutants proliferate normally and survive until the pupal stage^9^. To determine the relative contribution of E2f1 and E2f2 in muscle, each E2F family member was inactivated singularly, and the impact on myogenesis was investigated. Since *E2f1* mutants are lethal at larval stages, we used *UAS-E2f1-RNAi*^37^. As expected, depletion of E2f1 in proliferating myoblasts with *Mef2-GAL4* significantly impaired proliferation and resulted in early pupal lethality before muscle differentiation. Therefore, E2f1 was depleted specifically in the post-fusion indirect flight muscles with a late myogenic *Act88F-GAL4* driver^17^. The efficiency of E2f1 depletion was confirmed using qRT–PCR (Fig. 8a).

Transverse sections of thoraces of *Act88F>E2f1-RNAi* pharates were co-stained with Phalloidin and β-PS integrin antibody to reveal the organization of the DLMs. The depletion of E2f1 resulted in the reduced overall size of DLMs, while the overall structure and the number of DLMs remained unaffected (Fig. 8a). The area of DLM4 cross-sections, as well as DLM3, was significantly reduced in *Act88F>E2f1-RNAi* (Fig. 8a), which was highly reminiscent of the muscle defect in *Mef2>Dp-RNAi* (Fig. 3d). Notably, the analysis of DLM sagittal sections revealed an altered myofibril assembly (Fig. 8b), like in Dp-depleted muscles (Fig. 5a–c). Importantly, *Act88F>E2f1-RNAi* adult flies were severely impaired in their ability to fly, implying dysfunctional flight muscles (Fig. 8c). In this test, adult flies with fully functional flight muscles were able to fly to the highest region of the 1,000-ml cylinder when flipped into the column, while flightless flies usually fall to the bottom of the cylinder^19^. The relative distribution of flies over the height of the cylinder and the median distribution are shown in Fig. 8c. In contrast, the loss of *E2f2* had no effect on muscle size, myofibril width or the ability to fly (Fig. 8d–f).

To understand why the inactivation of E2f1 exerts a stronger effect on muscle morphology and function than the loss of E2f2, we evaluated the expression of myogenic genes in the flight muscles of each genotype. In agreement with the muscle defects observed above, the expression of myogenic genes was strongly deregulated in the absence of E2f1 (Fig. 8g). Depletion of E2f1 resulted in the downregulation of *Tm2*, *Act88F* and *Mlc2* genes. Interestingly, we observed higher levels of the myogenic regulators *Mef2*, *how* and *salm* that regulate the expression of muscle genes. This may explain why some structural genes were expressed at normal or higher levels in E2f1 depletion. Unlike E2f1, the loss of E2f2 exerted a much weaker effect on the myogenic transcriptional programme (Fig. 8h). Although some of the muscle-specific genes were slightly downregulated in the flight muscles of the *E2f2* mutant, none of the changes were statistically significant.

Collectively, these results suggest that E2f1 provides greater contribution to adult skeletal muscle development than E2f2.

## Discussion

**Figure 9:**
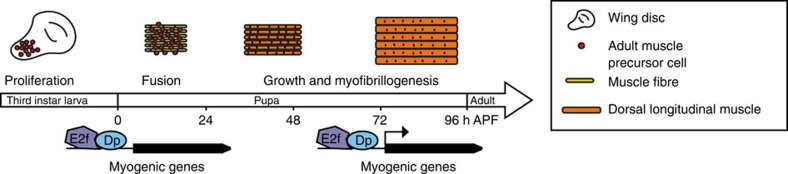
Model of myogenic gene expression regulation by E2F/Dp during *Drosophila* development. The adult myoblasts are localized in the adepithelial layer of wing discs. The myoblasts proliferate through larval development and fuse to the nascent muscle fibres in the thoracic cavity of pupae. On completion of fusion (∼36 h APF), the developing DLMs significantly increase their size, and both sarcomere units and myofibrils are assembled. Although E2F/Dp complexes occupy promoters of myogenic genes throughout development, they are needed only within a narrow temporal window during muscle growth to directly control the expression of several muscle genes. This novel role for E2F in late muscle differentiation is necessary and sufficient for adult fly viability.

In this report, we addressed a long-standing question in the E2F field: why do E2F-deficient animals die? Our goal was to understand what is the essential function of E2F in animal development. By expressing a *Dp* transgene in a spatially restricted pattern in *Dp* null mutant animals, we identified adult skeletal muscles as a critical tissue in which E2F function is necessary and sufficient for animal viability. Surprisingly, E2F requirement was restricted to late myogenesis when muscles undergo extensive growth. E2F occupies the promoters of muscle-specific genes, and their expression is markedly reduced in the absence of E2F, suggesting that E2F is directly involved in the control of myofibrillogenesis (Fig. 9). Thus, the role of E2F in regulating skeletal muscle growth is essential for animal viability.

Previous genome-wide studies in *Drosophila* revealed two broad categories of E2F target genes^6^,^7^,^8^. One group is regulated primarily by E2f1 activation and consists of cell cycle genes. Another group comprises genes related to gametogenesis, development and differentiation. These targets are regulated exclusively by E2f2-dependent repression, while E2f1 is never present on these targets. Therefore, the role of *Drosophila* E2F in differentiation was thought to prevent the inappropriate expression of differentiation programmes in cycling cells. Here we show that E2f1 has an important function in late muscle differentiation, where it controls the expression of muscle-specific genes.

Even though both E2f1 and E2f2 can be detected using ChIP on myogenic genes at different developmental stages, E2f1 is more important than E2f2 during muscle growth. This conclusion is supported by the strong impact of E2f1 knockdown on the size of the indirect flight muscles, and the poor performance of E2f1-depleted muscles in the flight ability test. In contrast, the *E2f2* mutation did not affect muscle size and function nor myogenic gene expression. These observations point to the functional importance of E2f1 during myogenic differentiation. However, we noted that *Act88F>E2f1-RNAi* results in a less severe phenotype than the knockdown of Dp with *Mef2-GAL4*. The differences in the severity of the phenotypes could be due to the knockdown efficiency of E2f1 and Dp, which is always a concern with any RNAi experiment, and the different strength, tissue specificity and timing of expression of two GAL4 drivers. Alternatively, this difference may reflect a partial redundancy between E2f1 and E2f2 since Dp depletion functionally inactivates both E2fs. In agreement, the knockdown of E2f1 exerted less severe effect on target gene expression in comparison with Dp depletion; thus, E2f2 could partially compensate for the loss of E2f1. Such an interpretation also parallels the relationship between the mammalian activator E2f3a and the repressor E2f4 during neuronal differentiation. In neural precursor cells (NPCs), both E2f3a and E2f4 bind and regulate the expression of differentiation genes; however, only E2f3 and not E2f4-deficient NPCs exhibit differentiation defects^38^,^39^.

The knockdown of Dp in the myogenic lineage revealed that the major defect associated with the loss of E2F function is the dramatic reduction in the size of the adult skeletal muscles. The most obvious explanation is that this is due to the reduced proliferation of Dp-deficient myoblasts, which would decrease the pool of myoblasts available for the formation of myotubes. A reduction in the number of myoblasts was shown to result in smaller muscles^24^,^40^,^41^. However, Dp-depleted myoblasts proliferated normally within the adepithelial layer in the wing imaginal disc, and there was no clear reduction in the number of migrating Dp-depleted myoblasts during fusion or in the number of nuclei in myotubes after the completion of fusion. The apparent lack of proliferation defect is in agreement with the normal packing density of myofibrils in Dp-depleted muscles, since reducing the number of myoblasts would result in loosely packed myofibrils^24^. In addition, altered myoblast proliferation was shown to reduce the total number of DLMs^24^,^40^,^41^, which was not the case in Dp-depleted muscles. Despite the lack of proliferation defects, we noted a small decrease in the number of nuclei in fully differentiated *Dp*-depleted fibres at 96 h APF, indicating that some nuclei are eliminated during muscle growth. However, the reduction in the number of nuclei does not precede the muscle growth defect, and therefore it is likely to be a secondary event. Thus, the knockdown of Dp does not interfere with the normal progression of development, and E2F function is dispensable for cell proliferation in flight muscle development.

During late myogenesis, muscle volume increases because of the continuous addition of filaments to myofibrils and accompanied muscle growth. These processes rely on high levels of expression of muscle-specific structural proteins. How the expression of late myogenic genes is maintained in adult muscles is not completely understood. The Cf2 transcription factor was previously linked to the control of structural gene expression; however, it acts as a repressor in adult flight muscles^32^. Other factors that may operate at this point to maintain muscle gene expression have not yet been identified, but might include Salm, Exd, Hth and the alternative splicing regulator Arrest, which are all involved in muscle fibre specification^42^,^43^,^44^,^45^. One implication of our work is that E2F contributes to the high level of expression of late muscle genes, and this contribution is needed to support myofibrillogenesis and muscle growth (Fig. 9). Among myogenic E2F targets are the muscle structural genes, including *Act88F*, *Mhc* and *fln*. Intriguingly, myofibrils were shown to be thinner in *Act88F* and *Mhc* heterozygotes, suggesting that even small changes in the levels of structural proteins, as found in Dp-depleted muscles, alter the stoichiometry of contractile proteins and could affect myofibril assembly^46^,^47^. Consistently, the sarcomere structure was less pronounced and the myofibril width was reduced in Dp-depleted muscles, indicating the presence of subtle defects in myofibril assembly. However, E2F is detected only on a subset of promoters of downregulated myogenic genes, suggesting that some of the transcriptional changes observed in *Mef2>Dp-RNAi* could be indirect. This could be the consequence of the altered expression of the key myogenic regulators, such as *Mef2*, *how* and *Lmpt*, which are misexpressed in Dp-depleted muscle and are direct targets of E2F.

The size of the indirect flight muscles also depends on mitochondrial fusion^24^. E2F/Dp was previously shown to regulate the expression of mitochondria-associated genes in *Dp*-mutant animals. Therefore, Dp-dependent mitochondrial morphology defects could also account for the muscle growth phenotype in Dp-deficient adult skeletal muscles.

An unexpected finding of our work is that the lethality of E2F-deficient animals is largely due to the requirement of E2F in adult skeletal muscles. Thus, during *Drosophila* development the essential function of E2F is not to control cell proliferation but rather to regulate terminal differentiation by engaging a myogenic transcriptional programme. Intriguingly, mammalian E2F factors were shown to regulate differentiation of various cell types by promoting tissue-specific transcriptional programmes. During myogenic differentiation, E2f3 is recruited to the promoters of muscle-specific genes and plays a critical role in their regulation^48^. In NPCs, E2f3 and E2f4 govern cell fate decisions and directly regulate the expression of differentiation genes^38^. Thus, the role of E2F in the regulation of tissue-specific transcriptional programmes is highly conserved, and the relevance of our findings extends beyond the fly model.

## Methods

### Fly stocks

The lines *1151-GAL4* (refs 15, 16, 17), *Act88F-GAL4* (ref. 17) and *P{twi-βgal}* were provided by Richard. M. Cripps. The stock *P{PTT-un1}slsZCL2144* characterized as a Z-band marker in the indirect flight muscles^49^ expresses a GFP-tagged Sls protein^50^. The line *weeP26* produces a Mhc-IFM19 splice variant fused to GFP^51^. The stock *vg*^*AME*^*-lacZ* was used to mark the developing indirect flight muscles^26^. The stock *P{PTT-GA}DpCA06954* (ref. 22) from the Carnegie collection, here annotated as *Dp*^*GFP*^, contains a GFP-expressing protein trap insertion. The expression of the GFP::Dp fusion protein was confirmed using western blot analysis (see details in Supplementary Fig. 2B). The *P{UAS-Dp.D}* stock used to overexpress Dp was recombined out from *P{UAS-E2f1.N}3B,P{UAS-Dp.D}1-4b/TM6B,Tb* (refs 52, 53). The *Dp*^*−/−*^ null mutant, denoted here as *Dp*^*a3*^/*Df(2R)Exel7124*, is a *trans*-heterozygote between the *Dp*^*a3*^ null allele of *Dp* (refs 5, 10) and the deficiency *Df(2R)Exel7124*, which deletes the entire *Dp* gene. The stocks *P{UAS-mito-HA-GFP.AP}*, which expresses GFP with a mitochondrial import signal, *P{UAS-GFP.nls}*, which expresses GFP with a nuclear localization signal, the GAL4 drivers *P{GawB}how24B*, *P{GAL4-Mef2.R}*^54^, *P{Mhc-GAL4.F3-580}*^32^, *P{GAL4-twi.G}*, *P{GAL4-twi.2xPE}*, *P{GAL4-arm.S}*, *P{GawB}AB1*, *P{GawB}c729*, *P{GawB}34B*, *P{r4-GAL4}*, *P{GawB}elavC155*, *P{nSyb-GAL4.P}*, *P{GawB}D42*, *P{GawB}insc*^*Mz1407*^, *P{GawB}tey*^*5053A*^ and *Act5C-GAL4*, were obtained from Bloomington Drosophila Stock Center (Bloomington, IN, USA). The line *rP298-GAL4* (ref. 55), a GAL4 insertion in the *kirre* locus (also known as *Duf*) was a gift from Mary K Baylies. The stocks *P{tinC-Gal4.Δ4}* and *P{bap3-GAL4}* were kindly provided by Manfred Frasch^56^,^57^. Both *P{Hand4.2-GAL4}* and *P{GMH5-GAL4}*^58^ stocks were a gift from Rolf Bodmer. The line *UAS-Dp-RNAi* was obtained from the library RNAi-GD (ID 12722) at the Vienna Drosophila Resource Center (Vienna, Austria). The gene expression phenotype was confirmed using *P{TRiP.JF02519}attP2* from TRiP at Harvard Medical School. The lethality phenotype using *Mef2-GAL4* driver was confirmed with *UAS-Dp-RNAi*^*4654R-2*^ from NIG-Fly Stock centre and *P{TRiP.HMS00245}attP2* from TRiP. The lines *{UAS-E2f1-RNAi}*^37^, E2f2^c03344^ and E2f2^76Q1^ (refs 9, 59) were used. The stocks *P{VALIUM20-mCherry}attP2* and *P{UAS-mCherry.VALIUM10}*attP2 from TRiP collection were used as control and obtained from Bloomington Drosophila Stock Center. All fly crosses were made at 25 °C in vials containing standard cornmeal-agar medium, except for the crosses containing *Act88F-GAL4* driver, which were kept at 29 °C during development.

### Fly viability experiment

Fly viability was assessed by collecting and transferring third instar larvae to fresh vials at 25 °C. The total number of larva, pupa, as well as pharate pupa, and adult flies able to eclose out of the pupal case was scored over time. The pupal developmental stages were assessed by following markers of metamorphosis as described in ref. 60. Images of 2- to 5-day-old adult flies were taken with the microscope Zeiss Discovery V8, × 0.63. In the case of the rescue experiments, at least 65 flies per genotype were scored in three independent experiments, except for the rescue using *1151-GAL4* driver, which was performed twice. The genotypes were confirmed using PCR and sequencing in the rescue experiments using *Mef2-GAL4* and *1151-GAL4* drivers in the *Dp*-mutant background. The viability experiment using different GAL4 drivers and *Dp-RNAi* was scored at least twice if Dp knockdown induced lethality.

### Flight test

The test of flight ability was adapted from ref. 19. Adult females were collected on eclosion and recovered at 25 °C for 4–6 days. A 1,000-ml cylinder was coated with mineral oil. Flies were flipped to the top of the column. Flies able to fly land on the top section of the column, while flightless flies fall to the bottom of the cylinder. The landing spot along the height of the cylinder was scored for each fly, and the frequency of landing spots per height of the cylinder was plotted. Each experiment was carried out three times. At least 20 flies per genotype were tested in each replicate.

### Histology

Pharate animals were removed from their pupal case at 96 h APF and were fixed with 4% formaldehyde at 4 °C overnight. Thoraces were embedded in paraffin, and sections of 8-μm thickness were cut using a microtome^61^. Haemotoxylin and eosin staining was performed using standard procedures. Images were taken using the bright-field microscope Zeiss ApoTome. At least 16 flies per genotype were scored.

### Immunofluorescence and confocal microscopy

Tissues were dissected and immediately fixed in 4% formaldehyde in PBS for 30 min, permeabilized in 0.3% Triton X-100 in PBS twice for 10 min each and blocked in 2% bovine serum albumin (BSA) with 0.3% Triton X-100 in PBS for 60 min. Samples were incubated with antibodies overnight at 4 °C in 2% BSA and 0.1% Triton X-100 in PBS. After washing four times for 10 min each in 0.1% Triton X-100 (in PBS), samples were incubated with appropriate Cy3- or Cy5-conjugated anti-mouse and anti-rabbit secondary antibodies (Jackson Immunoresearch Laboratories, used at 1:300) for 90 min in 10% normal goat serum and 0.1% Triton X-100 in PBS. After washing with 0.1% Triton X-100 (in PBS), tissues were stored in glycerol with antifade, and then mounted on glass slides. All steps were performed at room temperature, unless otherwise stated. The DLMs of pupa staged at 21 h APF were dissected as in ref. 62, fixed for 20–30 min, and washed in PBS with 0.1% Triton X-100. Thoraces of 2- to 5-day-old adult or pharate pupa staged at ∼96 h APF were fixed for 30 min in relaxing buffer. Thoraces were bisected in the sagittal plane, and were then fixed for an additional 15 min as described in ref. 63. In the case of transverse plane sectioning, flies were snap-frozen in liquid nitrogen and cut twice with a razor. The transverse sections were fixed for 1 h (ref. 24). For larval body wall musculature staining, larva was dorsally opened, pinned in a Sylgard dish and fixed for 20 min. A minimum of five to eight animals per genotype was dissected per experiment, except for the experiment that required quantitative analysis, in which case the staining was carried out two or three times.

The primary antibodies were mouse anti-β-gal (40-1a, 1:200, Developmental Studies Hybridoma Bank (DSHB)), mouse anti-β PS-integrin (CF.6G11, 1:50, DSHB), rabbit polyclonal anti-Dp (#212, 1:300)^7^ and rabbit anti-Mef2 (1:1,000)^64^. Rhodamine–phalloidin or fluorescein isothiocyanate–phalloidin were used to counterstain and stain thin filaments, and 4,6-diamidino-2-phenylindole for nucleus staining.

Fluorescent images were acquired with the laser-scanning confocal microscope (Zeiss LSM Observer.Z1) using × 20/0.8, × 40/1.2 and × 100/1.45 objectives. Images were processed using Photoshop (Adobe Systems). All images are confocal single-plane images and are oriented anterior to the left, unless otherwise stated. Only representative images are shown.

### Quantitative and statistical analysis

The total number of myoblasts in the adepithelial cells of the wing disc in late third instar larvae (120 h after egg-laying) was determined by marking the adult myoblast with *twi-lacZ* and Mef2 antibodies. Images were taken with confocal microscopy in a z-stack to cover all nuclei in the adepithelial layer, and then were projected into a single plane. The area covered by the adult myoblasts, as well as the disc area, was quantified using the ImageJ software. The plugin Image-based Tool for Counting Nuclei was used to determine the myonuclear number in the myoblast-positive area of the adepithelial layer. A minimum of 10 wing discs per genotype was scored.

The density and size of nuclei at 21 and 40 h APF were determined by staining nuclei of both myoblasts and developing DLMs using Mef2 antibody. Both the number and area of nuclei were measured using the analyse particle tool of the ImageJ software. The area of the selected region of interest was measured to calculate nuclear density. Two or three independent experiments were conducted per time point. A minimum of eight animals per genotype was scored.

The number of myofibrils was counted as the number of peaks of grey value (that is, Phalloidin intensity) over a distance of 20 or 40 μm using the plot profile tool from the ImageJ software. Three independent lines perpendicular to myofibril orientation were drawn over a set of myofibril clusters to count the number of peaks (that is, myofibrils). An average was calculated for each image (that is, hemithorax). Myofibril width was calculated as the distance divided by the total number of myofibrils within the selected region. At least six hemithoraces per genotype was quantified.

ImageJ was also used to quantify DLM 3 and DLM 4 cross-section area, as well as the myofibril cluster area in the transverse sections of indirect flight muscles, and the total area of the body wall muscle VL3. The numbers of muscle fibres, myofibrils and nuclei were manually counted per DLM cross-section in pharate animals. At least two independent experiments were conducted. A minimum of eight flies per genotype was quantified.

Images of the DLM sagittal section were blindly scored as either tubular or thin/fragmented regarding mitochondrial shape. At least 15 hemithoraces stained with mitoGFP were quantified per genotype. Two independent experiments were conducted.

Graphs were generated with the GraphPad Prism version 6.0 (Graphpad Software). The group means were analysed for overall statistical significance using the unpaired Student's *t*-test with Welch's correction, two-way analysis of variance and non-parametric analysis (Mann–Whitney and Kruskal–Wallis).

### RT–qPCR and ChIP–qPCR

Tissue was dissected as described above from at least three to five different animals. Total RNA was isolated with TRIzol (Invitrogen). Reverse transcription to measure standard mRNAs was performed using the SensiFAST cDNA Synthesis Kit (Bioline) according to the manufacturer's specifications. qPCR was performed with the SensiFast SYBR No-ROX Mix (Bioline) on a LightCycler 480 (Roche). The reference genes *RpL32*, *RpL30* and β*-tubulin* were validated as stable control genes^65^. Normalization was calculated using the geometric mean of these reference genes. Primer sequences are in Supplementary Table 2. At least three independent biological samples were collected for each genotype and developmental stage. Each sample was measured twice. Raw data for ChIP–qPCR presented in Fig. 7a are in Supplementary Tables 3 and 4, and raw data included in Fig. 7b are in Supplementary Table 5.

The degenerate E2F-binding sites NWTSSCSS^7^ and the *Drosophila* E2F motif TTGGCGC^6^ were identified from searches carried out with the Regulatory Sequence Analysis Tools^66^. The evolutionary conservation between *D. melanogaster* and *D. erecta* was considered to predict E2F-binding sites.

At least 15 animals staged at third instar larvae, and pupae at∼24 and 96 h APF were dissected out of their pupal cases and homogenized. Chromatin was extracted using homogenizer with 60 mM KCl, 15 mM NaCl, 4 mM MgCl_2_, 15 mM HEPES (pH 7.6), 0.5% Triton X-100, 0.5 mM dithiothreitol (DTT), 10 mM sodium butyrate and protease inhibitor cocktail (Complete, Roche) as in ref. 67. Samples were crosslinked for 15 min at room temperature in 1.8% formaldehyde, and 225 mM Glycine was added. Then, cells were lysed with 15 mM HEPES at pH 7.6, 140 mM NaCl, 1 mM EDTA, 0.5 mM EGTA, 0.1% sodium deoxycholate, 1% Triton X-100, 0.5 mM DTT, 0.1% SDS, 0.5% lauroylsarcosine and 10 mM sodium butyrate with protease inhibitor cocktail (Complete, Roche), and were sheared using a Branson 450 Sonifier. Chromatin was immunoprecipitated with rabbit anti-DP (#212 (ref. 7)), mouse anti-Rbf (DX3/DX5, ratio 1:1 (ref. 68)), rabbit anti-E2f1 (#210 (refs 7, 69), ratio 1:1) and rabbit anti-E2f2 antibodies (#79 (9)). Rabbit IgG (Sigma) was used as nonspecific antibodies. Complexes were pulled down with Protein G Dynabeads (Invitrogen), washed with lysis buffer four times, washed twice with TE (pH 8), eluted and decrosslinked overnight at 65 °C; RNA was degraded with RNase A (Sigma) for 1 h at 37 °C and protein was digested with proteinase K for 2 h at 50 °C. DNA was purified by phenol–chloroform extraction, followed by overnight ethanol precipitation. The input genomic DNA (before precipitation), as well as immunoprecipitated DNA, was quantified using qPCR as described here. Primer sequences are in Supplementary Table 2. A negative sequence site that does not contain any predicted E2F-binding sites, and the positive target genes, *Arp53D*, was used as controls (Supplementary Table 2). The protein enrichment was calculated as the percentage of immunoprecipitated DNA relative to input DNA (prior DNA precipitation) for each antibody. Data presented are relative to the negative binding site for each ChIP. Each sample was measured twice.

## Additional information

**How to cite this article:** Zappia, M. P. and Frolov, M. V. E2F function in muscle growth is necessary and sufficient for viability in *Drosophila*. *Nat. Commun.* 7:10509 doi: 10.1038/ncomms10509 (2016).

## Supplementary Material

Supplementary Information Supplementary Figures 1-4 and Supplementary Tables 1-5
