# Supplementary material for: E2F function in muscle growth is necessary and sufficient for viability in Drosophila
Source: Nat Commun. 2016 Jan 29;7:10509. doi: 10.1038/ncomms10509 (PMC4740182; doi:10.1038/ncomms10509)
Supplement: Supplementary Information — Supplementary Figures 1-4 and Supplementary Tables 1-5 [file ncomms10509-s1.pdf]

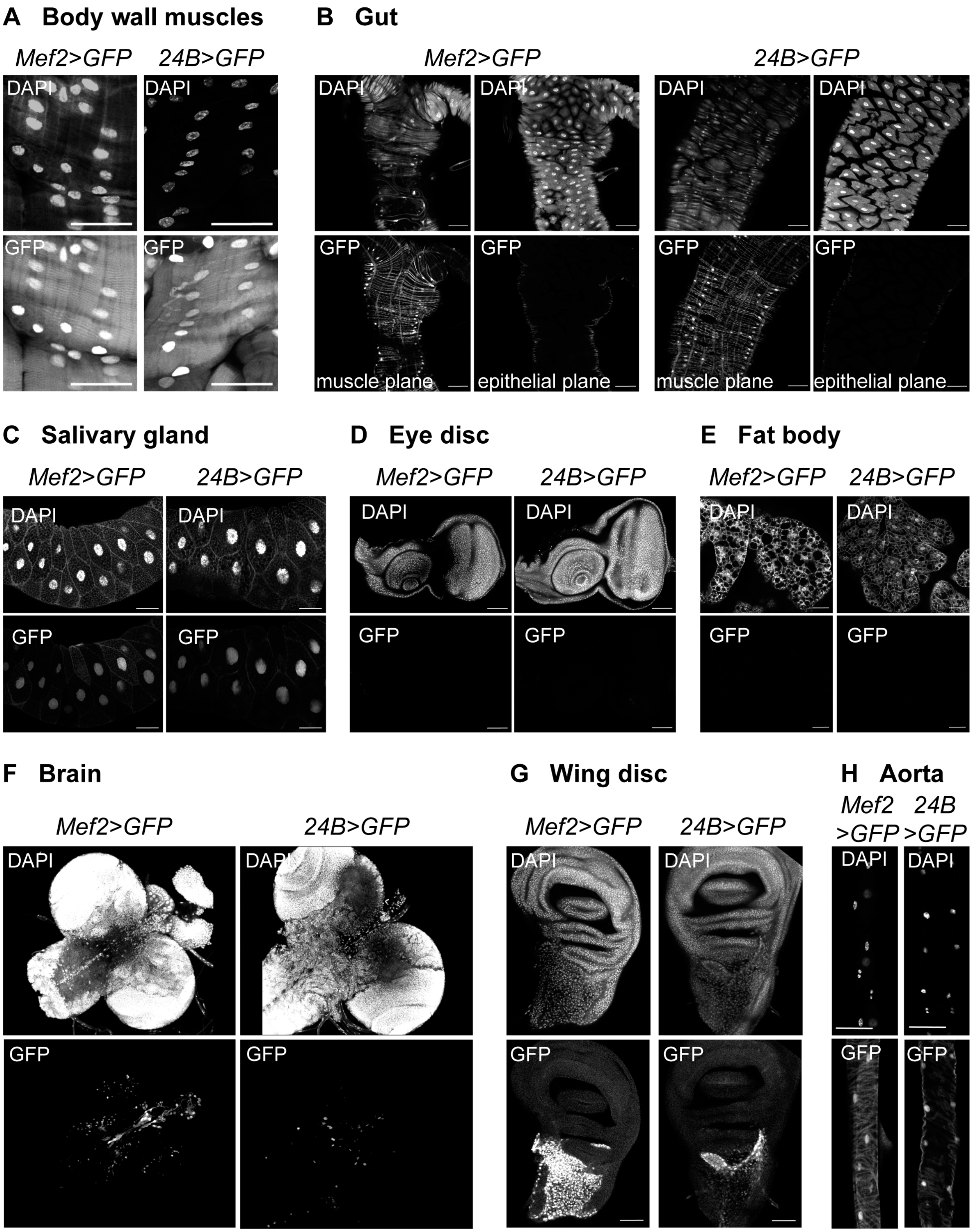

Supplementary Figure 1. ***Mef2-GAL4*** **and** ***24B-GAL4*** **are specific drivers for somatic, cardiac and visceral muscles**

Mef2-GAL4 and *24B-GAL4* are specific drivers for somatic, cardiac and visceral muscles, but leaked in salivary glands and some neurons of third instar larvae. GAL4 expression was reported using *UAS-GFP* (nuclear localization signal, bottom panel). Tissues were dissected and counterstained for DAPI (top panel). Confocal sections showed (**A**) body wall muscle, (**B**) gut at two different planes, muscle layer (left) and epithelial layer (right), (**C**) salivary gland, (**D**) eye disc, (**E**) fat body, (**F**) brain, (**G**) wing disc, and (**H**) cardiac tube of *Mef2-GAL4*;*UAS-GFP/* (left panel) and *24B-GAL4; UAS-GFP* (right panel). Scale bar is 50 µm. Images were taken and processed using the same parameters for comparative purpose.

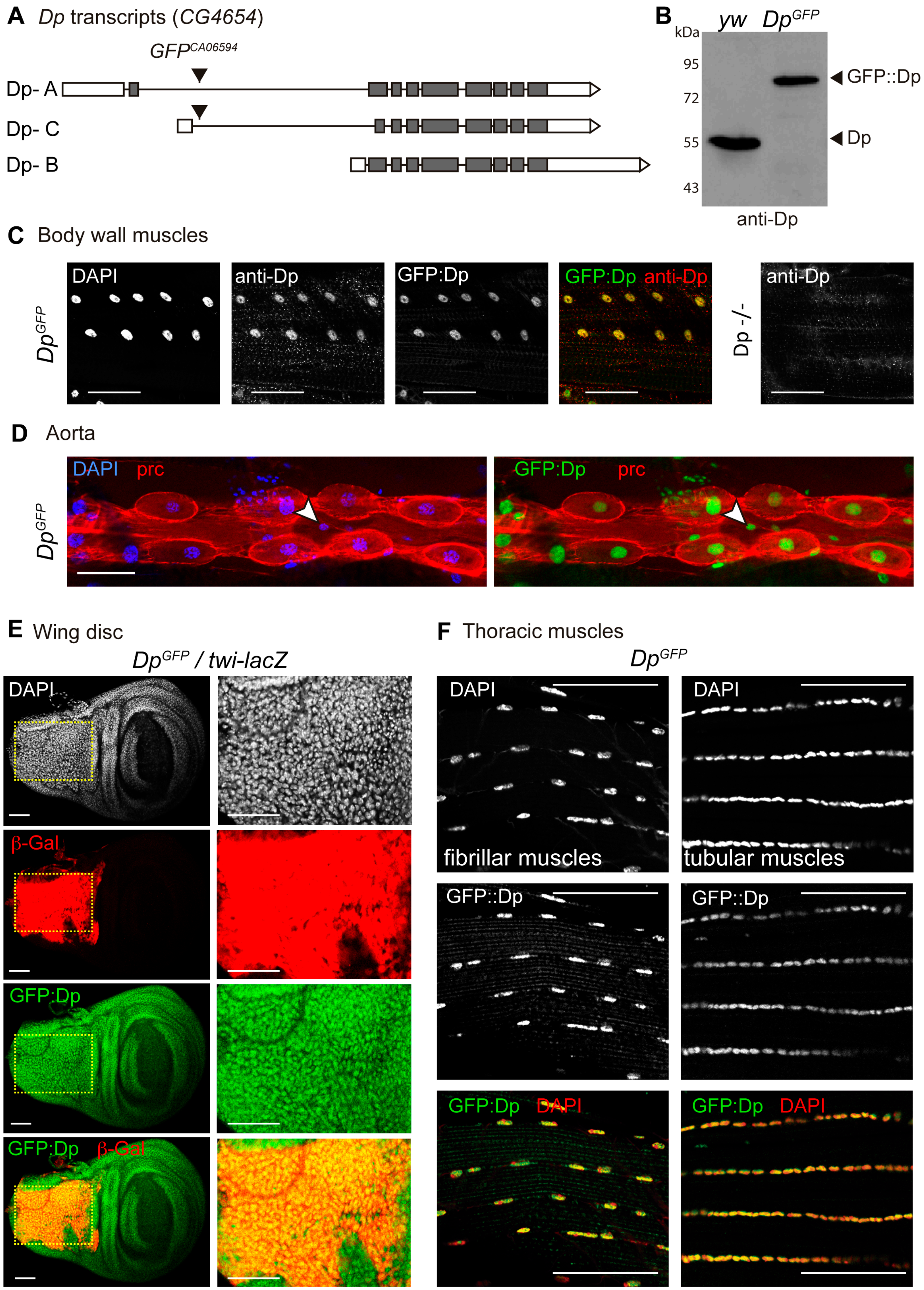

A Dp transcripts (CG4654)
GFP CA06594
Dp- A
Dp- C
Dp- B
B
kDa
yw
Dp GFP
95
72
55
43
GFP::Dp
Dp
anti-Dp
C Body wall muscles
Dp GFP
DAPI
anti-Dp
GFP:Dp
GFP:Dp anti-Dp
Dp -/-
anti-Dp
D Aorta
Dp GFP
DAPI prc
GFP:Dp prc
E Wing disc
Dp GFP / twi-lacZ
DAPI
β-Gal
GFP:Dp
GFP:Dp β-Gal
F Thoracic muscles
Dp GFP
DAPI
fibrillar muscles
tubular muscles
GFP::Dp
GFP:Dp DAPI

Supplementary Figure 2. Dp is localized in nuclei of larval and adult muscles

(**A**) Schematic diagram of the *Dp* transcripts (*Dp-A*, *Dp-B*, and *Dp-C*), which contains alternative transcription start sites. Exons and introns are indicated by boxes and lines, respectively. White boxes represent untranslated regions (UTR) and grey boxes highlight coding regions. The insertion location of the GFP-protein trap element (CA06594) is indicated by black arrowhead. Only transcripts *Dp-A* and *Dp-C* were predicted to contain GFP element and generate GFP-tagged proteins.

(**B**-**C**) Dp fusion to GFP was confirmed by western blot (**B**) and immunofluorescence (**C**). (**B**) A shift of approx 27 kDa was observed in *Dp[GFP]* compared to wild type (*yw*) larval homogenates using α-DP antibody. (**C**) Dp was localized in nuclei of body wall muscles at third instar larva. Dp::GFP colocalized with α-DP antibody and DAPI. The specificity of α-Dp antibody was analyzed by staining *Dp* null mutant (Dp^{a3}*/Df(2R)Exel7124*), which is lethal at pupal stage. Scale bar is 50 µm.

(D) Dp::GFP was detected in nuclei of cardiac cells (white arrowhead) and in pericardial cells, labeled with α-pericardin, in third instar larva cardiac tube. Scale bar is 50 µm.

(E) Confocal images of third instar larva wing disc showed Dp::GFP localized in nuclei of adult myoblasts labeled with *twi-lacZ* using β-Gal antibody (red). Magnifications of yellow dashed boxes are shown on the right panels. Scale bar is 50 µm.

(F) Dp::GFP was localized in nuclei of indirect flight muscles of adult flies. Fibrillar and tubular muscles are shown on the left and right panels, respectively. Scale bar is 50 µm.

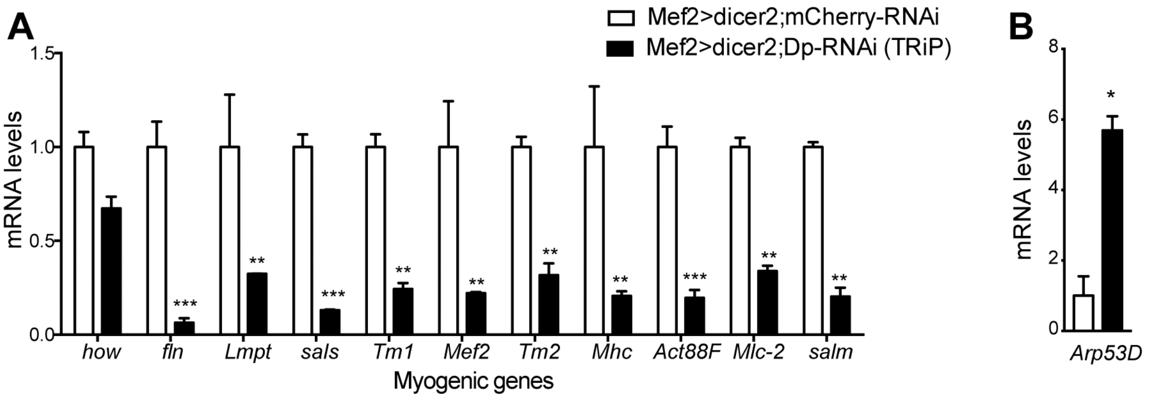

Supplementary Figure 3. Dp-depleted flight muscles displayed reduced expression of myogenic genes in pharates

An alternative *UAS-Dp-RNAi* from TRiP collection was used to knockdown Dp expression.
(A) Gene expression was quantified by RT-qPCR in indirect flight muscles of pharate pupa. Muscle genes are *held out wings* (*how*), *flightin* (*fln*), *Limpet* (*Lmpt*), *sarcomere length short* (*sals*), *Tropomyosin 1* (*Tm1*), *Myocyte enhancer factor 2* (*Mef2*), *Tropomyosin 2* (*Tm2*), *Myosin heavy chain* (*Mhc*), *Actin 88F (Act88F), Myosin light chain 2* (*Mlc2*), and *spalt major* (*salm*). Mean ± SEM, n=2 independent samples, two-way ANOVA, ** $p < 0.01$, *** $p < 0.001$.

(**B**) *Arp53D* gene expression. Mean ± SEM, n=2 independent samples, *t*-test with Welch's correction, * $p < 0.05$.
Genotypes are *Mef2-GAL4/UAS-mCherry-RNAi* (white bar) and *UAS-Dp-RNAi TRiP;Mef2-GAL4* (black bar).

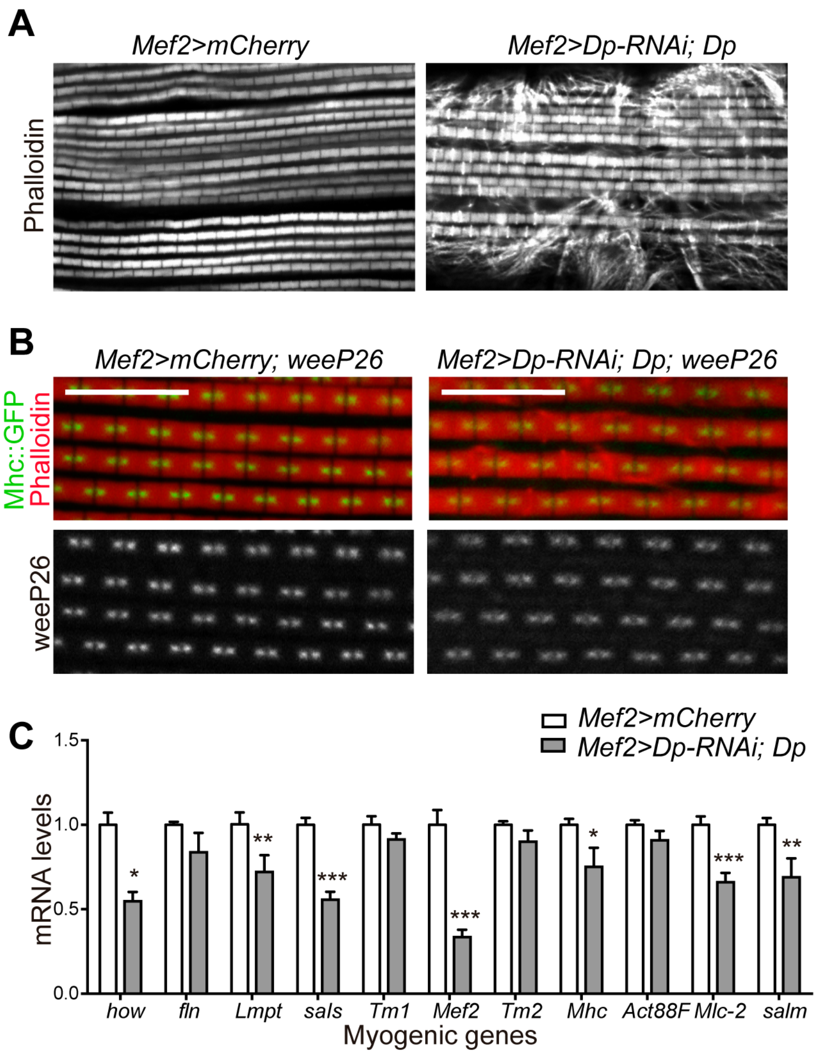

Supplementary Figure 4. Myofibril assembly is partially rescued in the few adults *Mef2>Dp-RNAi; UAS-Dp*

(**A**) Myofibrils at the edge of the Dorsal Longitudinal muscles are slightly defrayed in the partially rescued adult flies, thus indicating a mild yet persistent defect in myofibril morphology. Confocal images of DLMs stained with Phalloidin (red) in sagittal view.

(B) Both the reduced myofibril width and the defect in labeling of sarcomeric marker were rescued in partially rescued adult flies. Confocal images of DLMs in a sagittal view stained with Phalloidin (red) in a GFP-tagged splice variant Mhc-IFM19 background (*weeP26*), to mark A-band structure. Scale is 5 µm.

(C) The expression of whole set of myogenic genes was not fully restored in the partially rescued adult flies. Muscle genes are *held out wings* (*how*), *flightin* (*fln*), *Limpet* (*Lmpt*), *sarcomere length short* (*sals*), *Tropomyosin 1* (*Tm1*), *Myocyte enhancer factor 2* (*Mef2*), *Tropomyosin 2* (*Tm2*), *Myosin heavy chain* (*Mhc*), *Actin 88F (Act88F), Myosin light chain 2* (*Mlc2*), and *spalt major* (*salm*). Mean ± SEM, N =3 independent samples, two-way ANOVA, * $p < 0.05$, ** $p < 0.01$, *** $p < 0.001$.
Genotypes are *Mef2-Gal4/UAS-mCherry* (left panel, white bar), and *UAS-Dp-RNAi;Mef2-GAL4/UAS-Dp* (right panel, grey bar).

Supplementary Table 1: Phenotypes associated with knockdown of Dp by RNAi with Gal4 drivers

**GAL4 driver**	**GAL4 tissue expression**	**GAL4 expression developmental stage**	**Phenotype crossed to UAS-Dp-RNAi**
Act5C	Ubiquitous	Throughout dev.	Lethal
elavC155	Pan-neuronal	Throughout dev.	Viable, mild rough eye
nSyb	Pan-neuronal	Throughout dev.	Viable
Mz1407	Pan-neuronal	Throughout dev.	Semi-viable adults (30 % pharate)
D42	Motor neurons	Throughout dev.	Viable
arm.S	Epithelia	Throughout dev.	Viable
AB1<br>***c729***<br>***34B***	Salivary glands	Embryonic and larval dev.	Viable
r4	Fat body	Larval dev.	Viable
twi.G<br>***twi.2xPE***	Mesoderm	Embryonic dev.	Viable
24B	Mesoderm	Embryonic dev.	Lethal (pupa)
	- Precursors of somatic muscles, visceral muscles and cardiac cells<br>- All muscles	Embryonic and larval dev.	
	Adult muscle precursor cells	Adult myogenesis	
Mef2.R	Somatic, visceral and cardiac muscles	Embryonic and adult myogenesis	Lethal (pharate)
rP298	Progenitors and founders of somatic and visceral muscles	Embryonic dev.	Semi-lethal adults (54% pupa)
	- Thoracic and abdominal adult muscle founder cells<br>- Developing adult muscles	Adult myogenesis	
1151	- Adult muscle precursors<br>- Developing adult muscles<br>- Leg tendon cells and precursors	Adult myogenesis	Semi-lethal adults (74% pharate)<br>Flight/jump defect
Mhc.F3-580	Indirect Flight muscles at the onset of myofibrillogenesis (40 h APF at 22 °C) throughout adult	Late adult myogenesis	Flight/ jump defect
Act88F	Post-fusion Indirect Flight muscles throughout adult	Late adult myogenesis	Flight/ jump defect
bap3	Trunk visceral mesoderm	Embryonic dev.	Viable
5053A	Longitudinal musculature of the midgut	From end of embryogenesis throughout dev.	Viable
Hand4.2	Myocardium, epicardium, and visceral mesoderm	Embryonic dev.	Viable
GMH5	Myocardial cells	Larva and adult heart	Viable
tinC.Δ4	Cardiomyoblasts	Embryonic dev.	Viable

Supplementary Table 2. Primer sequence

Gene	Forward primer sequence	Reverse primer sequence	Application
Arp53D	TATAACAAATAAACGGGCACAGAAC	GCTCATGTTTCCAGGAGAGG	RT-qPCR
Him	AACCAGGTCCAGAATCACATCG	TGGTACTGCAGCTTGAAACG	RT-qPCR
twi	AAGCTCAGCAAGATCCAGACC	GAGCTGGCCGATCCATACG	RT-qPCR
Mef2	CGGATATCATGAGCCTCAACAC	CGTGGAACCATTGCTATTCTGC	RT-qPCR
blow	CGACTGTGTCGATAGCAAGG	TCGTAGAGCTGCTGATGTGG	RT-qPCR
sing	TACCATACGCCGATTCCATT	ACTGCGGATGTAGCCATACC	RT-qPCR
mbc	AAGACGCCAGATCCTCATGT	CTGAAGTGGAATCGGTGGTT	RT-qPCR
lmd	TAAAGCATTCTCGCGTTTGG	GATCCGTGTAGCGCTTGGT	RT-qPCR
ewg	TTATCACACTGCCGGATGGTAC	ACATCAGTGAGACTCTGAACGG	RT-qPCR
vg	ACCAAAGAAGAGGACCTCATCG	GGATGGCTGTGCGAGTGG	RT-qPCR
htl	AACGCATCGAAACCGTTCAC	TGGTGCGCTGTTTCTGTATC	RT-qPCR
stumps	AACAAGGTGGTTGCTCTGCT	ACTGCAGGGTGTAGGGATTG	RT-qPCR
Act88F	ATGGTGGGTATGGGTCAGAA	CTTCTCCATGTCGTCCCAGT	RT-qPCR
Mhc	AAGAACGACCTCGAGAACCAG	TCGGCCTTCTTCTTCTGCTG	RT-qPCR
fln	GGCAAAGAGGGACAAACAAC	ACTACGAGTGCTCATCCGTTC	RT-qPCR
TnT	CTTCACCATTGCCAAGAAGG	TTTGACAATGAGCTCCCACA	RT-qPCR
Actn	AGTACGGAGATGGCTACATGG	TGCGCAAATGGCTGTTACAC	RT-qPCR
Cf2	CACCACTCACCGTCAAGCTA	TGGCATAGACAGGCACAGAG	RT-qPCR
b-tubulin	ACATCCCGCCCCGTGGTC	AGAAAGCCTTGCGCCTGAACATAG	RT-qPCR
RpL32	TACAGGCCCAAGATCGTGAAG	GACGCACTCTGTTGTCGATACC	RT-qPCR
RpL30	GCAAATACTGCCTGGGCTAC	ACTTCAGTCTTGGCCAGCAT	RT-qPCR
COX5A	AAGGCCACCCTCTATCCCTA	CATCGTACACGGACTTCAGG	RT-qPCR
ND-B17.2	GTAGTGCATCCAGCCGTACC	CTACTTCTACGGCCGCAATC	RT-qPCR
Idh	GAGTAGGAGGCACCAGCAAC	TTTCCAATCCCAACCAGTTC	RT-qPCR
how	ATCTGTCCGATGACCTGCATG	TTAGCTCATCTTCGCCTTCGG	RT-qPCR
Lmpt-K	AGTGGCTGCCCTAAAACAAC	ATTGCTCCTCTGCTGCGATAG	RT-qPCR
sals	GCAAGCCATGAAGAAGAACCAG	TCGTCTTCGTCTAGCTTCATGG	RT-qPCR
Tm1	TGCAAGCGATGAAAGTCGAC	TCTGGATCTTCTTCTGCAGCTG	RT-qPCR
Tm2	TCCAAGATCATGGAGCTGGAG	TTCATCTCGCGCTTGAACTC	RT-qPCR
Mlc-2	TTCTCTGTGTTCTCCCAGAAGC	TTGTCGGCATCCATGAGTTG	RT-qPCR
salm	ACTACAGGAGCCACACCAAAG	ATGTGTTGCTTCAGGTTGCC	RT-qPCR
E2f1	ACCTGCTCAACGTGGATCTC	AATCTCGCGCGGCAATTTTG	RT-qPCR
How-650	GCGAAACAAAATGCCGCTTG	ATTAAGACACTGCGCTGTGG	ChIP-qPCR
Lmpt+100	CCAAAGCTGAAGCCAAAAGC	CACACACACAAAGACAGTCACTC	ChIP-qPCR
Sals-500	TTTGCCTTGTCAGCATCAGC	TAGCAATGGCCCGTTTAAGC	ChIP-qPCR
Mef2-220	TTCAGAGCGTCGAACTCCTTC	TGGAGTTCAAATCGCCCTTC	ChIP-qPCR
Tm2-130	ACGAGAATGCAGCCCAAAAG	TATGTGGAATGGCGCTTTCG	ChIP-qPCR
Tm1-IC-280	AGCGTTACCGTTAAGCAAGG	TTTTGCTGCGGCTGCTTATC	ChIP-qPCR
Fln -610bp	AACGGCGTGAAAACAATCGC	TACGAGCGCCTTATCGATGTG	ChIP-qPCR
Arp53D-108	TAGCTGCTTACGTATCGACTGC	TGTTGTGTGCTGTGTTCCAG	ChIP-qPCR
Negative site	TGTGTATGCCTTGCTTGCAC	TCTATGCACACGCTCTACTGAG	ChIP-qPCR

Supplementary Table 3. Raw data for E2f1 and IgG ChIP-qPCR of control and Mef2>Dp-RNAi at Pharate stage

		Fln		Tm2		Tm1-ic-300		how		Lmpt-bgh-100		sals-500		Mef2-fh-220		Negative site	
Genotype	Ab	Cp	Abs value	Cp	Abs value	Cp	Abs value	Cp	Abs value	Cp	Abs value	Cp	Abs value	Cp	Abs value	Cp	Abs value
Mef2>mCherry-RNAi	input 4% Dil 1/5	22.85	0.72	22.23	1.52	22.58	1.37	22.24	1.17	22.65	1.30	22.12	1.73	22.17	1.18	25.03	1.27
Mef2>mCherry-RNAi	input 4% Dil 1/5	22.82	0.73	22.20	1.55	22.67	1.29	22.15	1.24	22.63	1.31	22.15	1.68	22.14	1.21	25.04	1.26
Mef2>mCherry-RNAi	IgG	29.44	0.01	28.46	0.02	28.66	0.03	28.52	0.02	29.00	0.02	28.57	0.02	28.49	0.02	32.08	0.01
Mef2>mCherry-RNAi	IgG	29.44	0.01	28.22	0.02	29.11	0.02	28.34	0.02	28.72	0.02	28.61	0.02	28.61	0.01	32.23	0.01
Mef2>mCherry-RNAi	E2f1	27.69	0.03	27.58	0.04	27.44	0.06	25.68	0.12	26.82	0.07	26.57	0.08	27.34	0.03	30.99	0.03
Mef2>mCherry-RNAi	E2f1	27.89	0.03	27.67	0.03	27.34	0.06	25.57	0.13	26.82	0.07	26.52	0.08	27.48	0.03	31.11	0.03
Mef2>Dp-RNAi vdrc	input 4% Dil 1/5	23.78	0.39	23.80	0.50	24.33	0.44	23.58	0.48	23.98	0.52	23.55	0.63	23.55	0.46	26.04	0.66
Mef2>Dp-RNAi vdrc	input 4% Dil 1/5	23.78	0.39	23.86	0.48	24.32	0.44	23.55	0.50	24.02	0.51	23.59	0.61	23.53	0.46	26.11	0.64
Mef2>Dp-RNAi vdrc	IgG	29.25	0.01	28.73	0.02	29.42	0.02	28.51	0.02	29.41	0.01	28.69	0.02	28.53	0.02	32.04	0.01
Mef2>Dp-RNAi vdrc	IgG	28.91	0.01	28.48	0.02	28.95	0.02	28.53	0.02	29.03	0.02	28.67	0.02	28.92	0.01	31.68	0.02
Mef2>Dp-RNAi vdrc	E2f1	29.29	0.01	28.57	0.02	28.81	0.02	27.71	0.03	28.66	0.02	27.77	0.04	28.50	0.02	31.72	0.02
Mef2>Dp-RNAi vdrc	E2f1	29.22	0.01	28.58	0.02	29.29	0.02	27.55	0.04	28.19	0.03	28.24	0.03	28.72	0.01	31.33	0.02

Supplementary Table 4. Raw data for Rbf, Dp, E2f2 and IgG ChIP-qPCR of control and Mef2>Dp-RNAi at Pharate stage

		Negative site		Arp53D-prox3		Fln		how		sals-500		Mef2-FH-220		Lmpt-BGH-100		Tm1-IC-300		Tm2	
Genotype	Ab	Cp	Abs value	Cp	Abs value	Cp	Abs value	Cp	Abs value	Cp	Abs value	Cp	Abs value	Cp	Abs value	Cp	Abs value	Cp	Abs value
Mef2>mCherry-RNAi	10% - Dil 1/4	23.72	1.87	21.87	1.58	22.55	1.26	21.51	1.37	21.51	1.42	21.32	1.69	21.74	1.95	21.69	1.14	21.55	1.71
Mef2>mCherry-RNAi	10% - Dil 1/4	23.63	1.98	21.81	1.64	22.35	1.45	21.49	1.38	21.50	1.43	21.32	1.69	21.75	1.93	21.66	1.15	21.62	1.62
Mef2>mCherry-RNAi	IgG	30.24	0.03	27.76	0.03	28.90	0.02	27.55	0.03	27.89	0.02	27.68	0.02	27.96	0.03	28.01	0.02	27.61	0.02
Mef2>mCherry-RNAi	IgG	30.29	0.03	27.78	0.03	28.92	0.02	27.67	0.02	28.04	0.02	27.75	0.02	27.97	0.03	28.10	0.02	27.83	0.02
Mef2>mCherry-RNAi	Dp	29.28	0.05	22.31	1.18	25.03	0.24	22.64	0.65	24.12	0.23	24.51	0.19	23.86	0.46	23.05	0.47	26.74	0.05
Mef2>mCherry-RNAi	Dp	29.16	0.06	22.22	1.25	24.95	0.26	22.57	0.68	24.25	0.21	24.52	0.19	23.79	0.48	23.03	0.48	26.83	0.04
Mef2>mCherry-RNAi	E2f2	29.52	0.05	21.78	1.67	24.72	0.30	22.44	0.74	24.04	0.24	24.15	0.24	23.45	0.60	22.76	0.57	26.92	0.04
Mef2>mCherry-RNAi	E2f2	29.32	0.05	21.69	1.78	24.73	0.30	22.30	0.81	24.07	0.23	24.15	0.24	23.48	0.59	22.73	0.58	26.72	0.05
Mef2>mCherry-RNAi	Rbf1	30.58	0.02	22.51	1.03	25.92	0.14	23.34	0.41	25.30	0.10	25.27	0.11	24.62	0.27	23.83	0.28	27.73	0.02
Mef2>mCherry-RNAi	Rbf1	30.63	0.02	22.53	1.02	25.89	0.14	23.33	0.41	25.57	0.08	25.25	0.11	24.58	0.28	23.86	0.28	28.16	0.02
Mef2>Dp-RNAi	10% - Dil 1/4	23.76	1.83	21.92	1.53	21.72	2.19	21.68	1.22	21.47	1.46	21.45	1.55	21.64	2.10	22.03	0.91	21.69	1.55
Mef2>Dp-RNAi	10% - Dil 1/4	23.76	1.82	21.91	1.53	21.83	2.04	21.45	1.42	21.49	1.44	21.34	1.67	21.76	1.92	22.04	0.90	21.69	1.55
Mef2>Dp-RNAi	IgG	30.35	0.03	28.58	0.02	28.54	0.02	27.71	0.02	27.71	0.02	27.87	0.02	28.48	0.02	28.48	0.01	27.79	0.02
Mef2>Dp-RNAi	IgG																		
Mef2>Dp-RNAi	Dp	30.43	0.03	24.17	0.34	26.61	0.09	24.29	0.22	26.03	0.06	26.51	0.05	25.69	0.13	24.84	0.15	27.99	0.02
Mef2>Dp-RNAi	Dp	30.58	0.02	24.12	0.36	26.48	0.09	24.33	0.21	25.93	0.07	26.61	0.04	25.67	0.13	24.93	0.14	27.87	0.02
Mef2>Dp-RNAi	E2f2	30.56	0.02	24.34	0.31	26.43	0.10	24.57	0.18	26.08	0.06	26.48	0.05	25.71	0.13	24.72	0.16	27.92	0.02
Mef2>Dp-RNAi	E2f2	30.60	0.02	24.20	0.34	26.43	0.10	24.53	0.19	26.27	0.05	26.55	0.05	25.81	0.12	24.84	0.15	27.95	0.02
Mef2>Dp-RNAi	Rbf1	30.58	0.02	25.56	0.14	27.60	0.04	25.87	0.08	27.24	0.03	27.32	0.03	26.84	0.06	26.23	0.06	27.82	0.02
Mef2>Dp-RNAi	Rbf1	30.75	0.02	25.59	0.14	27.42	0.05	25.97	0.07	26.91	0.03	27.17	0.03	26.76	0.06	26.14	0.06	28.47	0.01

Supplementary Table 5. Raw data for Rbf, Dp, E2f2, E2f1 and IgG ChIP-qPCR of control animals at 3rd intard larvae, and pupa staged at 24 and 96 h APF

			Fln		Arp53d-prox3		Tm2		Tm1-ic-300		how		Lmpt-bgh-100		sals-500		Mef2-fh-220		Negative sit	
Genotype	Stage	Ab	Cp	Abs value	Cp	Abs value	Cp	Abs value	Cp	Abs value	Cp	Abs value	Cp	Abs value	Cp	Abs value	Cp	Abs value	Cp	Abs value
Mef2>mCherry-RNAi	L3	input 4% Dil 1/5	22.24	1.08	22.06	1.52	21.75	2.12	21.72	2.39	21.58	1.81	21.94	2.11	21.66	2.38	21.51	1.85	24.30	2.02
Mef2>mCherry-RNAi	L3	input 4% Dil 1/5	22.29	1.04	22.04	1.54	21.63	2.31	21.78	2.29	21.61	1.77	21.97	2.07	21.54	2.59	21.58	1.77	24.45	1.84
Mef2>mCherry-RNAi	L3	IgG	28.27	0.02	28.05	0.03	27.18	0.05	27.79	0.05	27.50	0.04	27.85	0.04	27.66	0.04	27.42	0.03	30.58	0.04
Mef2>mCherry-RNAi	L3	IgG	28.49	0.02	27.62	0.04	27.28	0.04	27.77	0.05	27.52	0.04	28.26	0.03	27.68	0.04	27.55	0.03	30.61	0.04
Mef2>mCherry-RNAi	L3	E2f1	26.99	0.05	27.19	0.05	27.33	0.04	26.87	0.08	24.26	0.31	25.97	0.13	25.89	0.13	26.79	0.05	30.93	0.03
Mef2>mCherry-RNAi	L3	E2f1	26.95	0.05	27.06	0.06	27.21	0.05	26.75	0.09	24.31	0.30	25.84	0.15	25.76	0.14	26.80	0.05	30.64	0.04
Mef2>mCherry-RNAi	L3	Dp	25.21	0.15	22.31	1.29	27.58	0.04	23.17	0.93	22.55	0.95	23.94	0.54	24.51	0.32	25.09	0.16	30.79	0.03
Mef2>mCherry-RNAi	L3	Dp	25.23	0.15	22.25	1.34	27.48	0.04	23.12	0.96	22.49	1.00	24.04	0.50	24.54	0.31	25.05	0.16	30.42	0.04
Mef2>mCherry-RNAi	L3	E2f2	24.65	0.22	21.50	2.20	27.60	0.04	22.58	1.37	22.18	1.22	23.56	0.70	24.17	0.41	24.65	0.22	30.58	0.04
Mef2>mCherry-RNAi	L3	E2f2	24.56	0.23	21.56	2.12	27.48	0.04	22.52	1.42	22.12	1.26	23.53	0.71	24.34	0.36	24.62	0.22	31.18	0.02
Mef2>mCherry-RNAi	L3	Rbf1	26.04	0.09	22.63	1.04	28.69	0.02	24.02	0.54	23.51	0.51	24.67	0.33	25.85	0.13	25.98	0.09	31.82	0.02
Mef2>mCherry-RNAi	L3	Rbf1	26.07	0.08	22.70	1.00	28.64	0.02	24.01	0.54	23.60	0.48	24.87	0.28	25.78	0.13	25.96	0.09	31.74	0.02
Mef2>mCherry-RNAi	P24	input 4% Dil 1/5	22.20	1.11	21.94	1.65	21.54	2.45	21.50	2.75	21.47	1.95	21.67	2.55	21.60	2.48	21.32	2.11	24.09	2.31
Mef2>mCherry-RNAi	P24	input 4% Dil 1/5	22.20	1.11	21.89	1.69	21.60	2.35	21.47	2.80	21.32	2.14	21.67	2.53	21.62	2.45	21.45	1.94	24.03	2.41
Mef2>mCherry-RNAi	P24	IgG	28.47	0.02	27.91	0.03	27.59	0.04	27.70	0.05	27.71	0.03	28.07	0.03	27.65	0.04	27.58	0.03	30.78	0.03
Mef2>mCherry-RNAi	P24	IgG	28.46	0.02	27.89	0.03	27.44	0.04	27.35	0.06	27.65	0.03	27.96	0.03	27.78	0.04	27.60	0.03	30.62	0.04
Mef2>mCherry-RNAi	P24	E2f1	26.51	0.06	26.70	0.07	26.72	0.07	26.18	0.13	24.18	0.33	24.97	0.27	25.94	0.12	25.95	0.09	29.87	0.06
Mef2>mCherry-RNAi	P24	E2f1	26.32	0.07	26.82	0.07	26.76	0.06	26.20	0.13	24.20	0.32	24.96	0.27	25.71	0.14	25.96	0.09	29.87	0.06
Mef2>mCherry-RNAi	P24	Dp	24.92	0.18	22.29	1.30	26.74	0.06	22.65	1.30	22.55	0.96	23.81	0.59	24.90	0.24	24.28	0.28	30.09	0.05
Mef2>mCherry-RNAi	P24	Dp	24.92	0.18	22.42	1.20	26.75	0.06	22.63	1.32	22.56	0.95	23.80	0.59	24.98	0.23	24.26	0.28	30.28	0.04
Mef2>mCherry-RNAi	P24	E2f2	24.51	0.24	21.53	2.16	26.69	0.07	22.09	1.88	22.18	1.22	23.61	0.67	24.89	0.25	23.84	0.37	30.22	0.05
Mef2>mCherry-RNAi	P24	E2f2	24.52	0.24	21.53	2.16	26.70	0.07	22.05	1.93	22.31	1.12	23.63	0.67	24.84	0.25	23.81	0.38	29.93	0.06
Mef2>mCherry-RNAi	P24	Rbf1	25.34	0.14	21.96	1.62	27.42	0.04	22.92	1.09	23.02	0.70	24.27	0.43	25.68	0.14	24.67	0.21	30.35	0.04
Mef2>mCherry-RNAi	P24	Rbf1	25.27	0.14	22.03	1.55	27.30	0.04	22.92	1.10	23.00	0.71	24.23	0.44	25.81	0.13	24.62	0.22	30.72	0.03
Mef2>mCherry-RNAi	P96	input 4% Dil 1/5	22.85	0.72	22.81	0.93	22.23	1.52	22.58	1.37	22.24	1.17	22.65	1.30	22.12	1.73	22.17	1.18	25.03	1.27
Mef2>mCherry-RNAi	P96	input 4% Dil 1/5	22.82	0.73	22.72	0.98	22.20	1.55	22.67	1.29	22.15	1.24	22.63	1.31	22.15	1.68	22.14	1.21	25.04	1.26
Mef2>mCherry-RNAi	P96	IgG	29.44	0.01	28.95	0.02	28.46	0.02	28.66	0.03	28.52	0.02	29.00	0.02	28.57	0.02	28.49	0.02	32.08	0.01
Mef2>mCherry-RNAi	P96	IgG	29.44	0.01	29.17	0.01	28.22	0.02	29.11	0.02	28.34	0.02	28.72	0.02	28.61	0.02	28.61	0.01	32.23	0.01
Mef2>mCherry-RNAi	P96	E2f1	27.69	0.03	27.90	0.03	27.58	0.04	27.44	0.06	25.68	0.12	26.82	0.07	26.57	0.08	27.34	0.03	30.99	0.03
Mef2>mCherry-RNAi	P96	E2f1	27.89	0.03	27.92	0.03	27.67	0.03	27.34	0.06	25.57	0.13	26.82	0.07	26.52	0.08	27.48	0.03	31.11	0.03
Mef2>mCherry-RNAi	P96	Dp	25.48	0.13	22.70	1.00	27.57	0.04	23.83	0.60	23.04	0.69	24.46	0.38	24.67	0.29	25.09	0.16	31.05	0.03
Mef2>mCherry-RNAi	P96	Dp	25.34	0.14	22.73	0.98	27.65	0.03	23.85	0.60	23.04	0.69	24.55	0.35	24.64	0.29	25.08	0.16	31.28	0.03
Mef2>mCherry-RNAi	P96	E2f2	25.44	0.13	22.52	1.12	27.95	0.03	23.29	0.86	22.90	0.76	24.18	0.46	24.68	0.29	24.96	0.17	31.02	0.03
Mef2>mCherry-RNAi	P96	E2f2	25.16	0.16	22.53	1.11	27.84	0.03	23.22	0.90	22.90	0.76	24.16	0.46	24.64	0.29	25.01	0.17	31.01	0.03
Mef2>mCherry-RNAi	P96	Rbf1	26.70	0.06	23.92	0.45	28.03	0.03	25.00	0.28	24.58	0.25	25.82	0.15	26.30	0.10	26.56	0.06	31.25	0.02
Mef2>mCherry-RNAi	P96	Rbf1	26.78	0.05	23.92	0.44	28.21	0.02	25.04	0.28	24.53	0.26	25.78	0.15	26.24	0.10	26.67	0.05	31.54	0.02
